# *OsAAP6* functions as an important regulator of grain protein content and nutritional quality in rice

**DOI:** 10.1038/ncomms5847

**Published:** 2014-09-11

**Authors:** Bo Peng, Huili Kong, Yibo Li, Lingqiang Wang, Ming Zhong, Liang Sun, Guanjun Gao, Qinglu Zhang, Lijun Luo, Gongwei Wang, Weibo Xie, Junxiao Chen, Wen Yao, Yong Peng, Lei Lei, Xingmin Lian, Jinghua Xiao, Caiguo Xu, Xianghua Li, Yuqing He

**Affiliations:** 1 National Key Laboratory of Crop Genetic Improvement and National Centre of Plant Gene Research (Wuhan), Huazhong Agricultural University, Hongshan District, Wuhan 430070, China; 2 Shanghai Agrobiological Gene Center, Shanghai 201106, China

## Abstract

Grains from cereals contribute an important source of protein to human food, and grain protein content (GPC) is an important determinant of nutritional quality in cereals. Here we show that the quantitative trait locus (QTL) *qPC1* in rice controls GPC by regulating the synthesis and accumulation of glutelins, prolamins, globulins, albumins and starch. *qPC1* encodes a putative amino acid transporter *OsAAP6*, which functions as a positive regulator of GPC in rice, such that higher expression of *OsAAP6* is correlated with higher GPC. *OsAAP6* greatly enhances root absorption of a range of amino acids and has effects on the distribution of various amino acids. Two common variations in the potential *cis*-regulatory elements of the *OsAAP6* 5′-untranslated region seem to be associated with GPC diversity mainly in *indica* cultivars. Our results represent the first step toward unravelling the mechanism of regulation underlying natural variation of GPC in rice.

## Abstract (web summary)

Grain protein content (GPC) contributes to the nutritional quality of cereals. Here, the authors show that the OsAAP6 quantitative trait locus in rice controls GPC by regulating the synthesis and accumulation of several grain storage proteins and starch.

## Graphical abstract

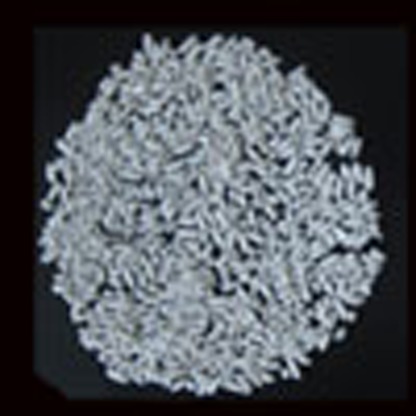

Enhancing nutritional quality of cereals is a means of improving human nutrition and health^1^. Rice, wheat, maize and barley contain different contents of grain protein, which is an important part of total protein in human food^2^. Nearly one-third of the world population currently suffers from protein malnutrition and some diseases are caused by poor-quality protein and lack of vitamins and other micronutrients^3^. Over 90% of rice grain consists of starch and proteins^4^,^5^ and grain protein content (GPC) is a key factor determining the nutritional quality^6^. Rice is generally recognized as having the lowest GPC of the cereal grains, but net protein utilization from rice is highest among the cereal grains^7^. Moreover, the demand for high-quality cereals as a source of protein is expected to increase dramatically in the near future^4^.

The amino acid composition in rice grain is relatively balanced and the grain proteins are divided into albumins, globulins, prolamins and glutelins^8^. GPC in rice grain is mainly constituted of glutelins and prolamins; glutelins have high nutritive value for the human diet^6^,^9^. Thus, GPC is an attractive target for enhancing nutritional quality of rice grain. As GPC in rice is determined by complex multigene families and highly polymorphic mixtures of polypeptides, even mutations in a few structural genes have little effect on GPC^8^,^10^,^11^. Interestingly, some genes not only regulate starch storage but also have effects on protein in the endosperm, for example, transgenic RNA interference (RNAi) targeting the *Opaque7* gene causes downregulation of GPC in both maize and rice, and the *FLOURY ENDOSPERM2* gene mutant results in reduced accumulation of glutelin^12^,^13^. Although *gpa1* regulates storage protein trafficking, GPC in a rice *gpa1* mutant is the same as in wild-type plants^14^. Many genes/regulators affecting GPC have been isolated from various mutants in cereals^12^,^13^,^14^,^15^,^16^ and numerous quantitative trait loci (QTLs) for GPC have been detected in genetic mapping populations^17^,^18^,^19^,^20^,^21^,^22^. However, no QTL underlying natural variation and controlling GPC in rice has been cloned, and little is known about how GPC is regulated quantitatively.

Our previous studies revealed a relatively strong QTL cluster controlling GPC^22^. Several loci within the cluster encode putative amino acid transporters^23^ and the RM472–RM104 region on the long arm of chromosome 1 exhibits the highest value (32.4%) of trait variation explained by one QTL among the clusters^22^. Here we report the cloning of the major QTL *qPC1* (a putative amino acid permease, *OsAAP6*) controlling natural variation in GPC by a map-based cloning strategy. We show through overexpression, complementation, RNAi and transmission electron microscopy studies that *OsAAP6* functions as an important regulator of GPC and nutritional quality in rice.

## Results

### Mapping the *qPC1* locus to a 6.7-kb region

**Figure 1:**
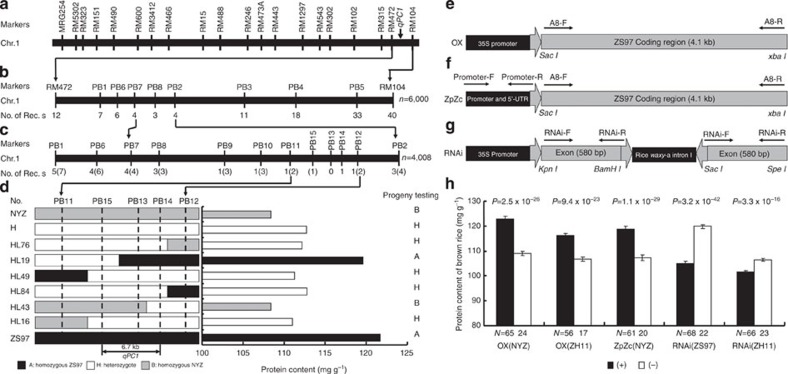
Map-based cloning of the *qPC1* QTL and grain protein content of transgenic plants in T_1_. (**a**) Location of the *qPC1* QTL on the genetic linkage map of chromosome 1. (**b**,**c**) Fine mapping of the *qPC1* region using two mapping populations, with 6,000 and 4,008 plants, respectively. No. of Recs, number of recombinants between *qPC1* and indicated molecular marker (**b**). Numbers in parentheses in **c** correspond to the numbers of recombinants in the **b**. (**d**) Genotypes and phenotypes of the recombinants. The phenotype of each recombinant was determined by progeny testing (Supplementary Table 2). (**e**) The *OsAAP6* coding region of ZS97 was inserted into the vector pCAMBIA1301S under control of the CaMV 35S promoter to prepare the overexpression construct (OX). Arrows represent the direction of PCR primers. (**f**) The 1.8-kb promoter fragment and 5′-UTR of *OsAAP6* from ZS97 with its own coding region was inserted into the vector pCAMBIA1301 to prepare the complementation construct (ZpZc). Arrows represent the direction of PCR primers. (**g**) The 580 bp cDNA fragment from the fourth exon of *OsAAP6* was inserted into the dspCAMBIA1301 vector to generate the RNAi construct. Arrows represent the direction of PCR primers. (**h**) Grain protein contents of transgenic plants in T_1_. N, number of plants; (+) and (−) indicate transgene-positive and transgene-negative plants, respectively. *P*-values were produced by two-tailed *t*-test. Error bars, s.e.m.

To identify the gene(s) underlying the *qPC1* QTL for GPC, we used 190 recombinant inbred lines (RILs) derived from a cross between Zhenshan 97 (ZS97, *Oryza sativa* L. *ssp. indica*) and Nanyangzhan (NYZ, *O. sativa* L. *ssp. japonica*). *qPC1* was mapped to the interval between markers RM472 and RM104 on the long arm of chromosome 1 (Fig. 1a). The allele from ZS97 contributed to increased GPC and total amount of essential amino acids^22^.

To fine map the *qPC1* locus, we backcrossed RIL-105 (one of the RILs containing the chromosome segment RM472–RM104 from NYZ and 78% of the genetic background of ZS97) three times (BC_3_) to ZS97. BC_3_F_1_ heterozygous plants were self-pollinated to develop two BC_3_F_2_ populations consisting of 6,000 (population 1) and 4,008 (population 2) individuals, and to establish a pair of near-isogenic lines (NILs), NIL(ZS97) and NIL(NYZ). Analysis of a BC_3_F_2_ population of 320 individuals derived by self-pollination of a BC_3_F_1_ heterozygote showed that GPC in heterozygous plants was significantly lower than in ZS97 homozygotes but higher than in NYZ homozygotes (Supplementary Table 1), indicating that the ZS97 high-protein content allele of *qPC1* was partially dominant.

Fifty-two recombinants between RM472 and RM104 were identified in population 1 and their genotypes at the *qPC1* locus were deduced by progeny testing. Using these data, we mapped *qPC1* to the interval between PB2 and PB7 (Fig. 1b). Seven recombinants between PB2 and PB7 were detected in population 2 (Fig. 1c). Using markers PB8–PB15, we identified six and two recombinant plants in the intervals PB11–PB12 and PB14–PB15 in the two populations, respectively (Fig. 1c,d). Each recombinant was further phenotyped by progeny testing to deduce the genotype of *qPC1* (for example, see Supplementary Table 2). The *qPC1* locus co-segregated with marker PB13, located between PB14 and PB15 (Fig. 1c,d).

To convert the genetic map location of *qPC1* into a physical map location, we placed the molecular markers onto the physical map of Nipponbare, the reference cultivar for rice genomics. As shown in Supplementary Fig. 1, the PB10–PB15 region was in the same order on the physical map of Nipponbare and on the genetic map derived from the ZS97** × **NYZ population (Fig. 1c). In the Nipponbare genome, the distance between PB14 and PB15 is 6.7 kb (Supplementary Fig. 1).

Analysis of the genomic DNA data revealed only one predicted gene (NCBI accession no. Loc_Os01g65670) in the 6.7 kb region and the gene has three predicted full-length complementary DNA (cDNA) sequence (AK121636, AK065286 and AK119529). The sequence of AK121636 includes the full-length cDNA sequence of AK065286 and AK119529, and all of them encode the same amino acid sequence. Thus, this gene (Loc_Os01g65670) was considered the candidate gene for *qPC1* (Supplementary Fig. 1). Alignment of the full-length cDNA sequence of AK121636 with the genomic sequence of Nipponbare indicated that *qPC1* encodes a putative amino acid transporter belonging to the APC superfamily and has a PF01490 consensus domain ( http://pfam.janelia.org/). Phylogenetic analysis of the qPC1 protein revealed that it is highly homologous to the amino acid permeases (AAPs) family (Supplementary Fig. 2). Interestingly, *qPC1* corresponds to *OsAAP6* (Loc_Os01g65670), which is highly expressed in seeds and belonging to *OsAAT* family in rice^23^.

### Confirmation of the *qPC1* QTL

Three transformation constructs were prepared to confirm the candidate gene (*OsAAP6* (ref. 23)) for the *qPC1* QTL. First, a construct for overexpression (OX) contained the coding region of *OsAAP6* from ZS97 (high GPC) driven by the CaMV 35S promoter inside the vector pCAMBIA1301 (Fig. 1e); second, a complementation construct (ZpZc) with the promoter and coding regions of *OsAAP6* from ZS97 inserted into pCAMBIA1301S (Fig. 1f); and third, an RNAi construct containing a 580-bp PCR fragment from the fourth exon of *OsAAP6* inserted into dspCAMBIA1301 in both the sense and antisense orientations (Fig. 1g). *Agrobacterium*-mediated transformation was used to introduce OX into NYZ (OX(NYZ)) and Zhonghua 11 (ZH11, OX(ZH11)), ZpZc into NYZ (ZpZc(NYZ)), and RNAi into ZS97 (RNAi(ZS97)) and ZH11 (RNAi(ZH11)).

All transgene-positive individuals of OX(NYZ), OX(ZH11) and ZpZc(NYZ) in the T_0_ generation had a higher GPC than transgene-negative individuals (Supplementary Fig. 3). Co-segregation analysis between the genotypes and phenotypes in T_1_ progenies confirmed these effects: all transgene-positive plants of OX(NYZ), OX(ZH11) and ZpZc(NYZ) had higher GPC than the untransformed controls (Fig. 1h), whereas lower GPC was observed for RNAi(ZS97) and RNAi(ZH11) in both T_0_ (Supplementary Fig. 3) and the T_1_ (Fig. 1h). Co-segregation analysis of genotypes and expression levels with phenotypes in T_2_ progenies confirmed that GPC was significantly (*P*<0.01) and positively correlated with expression level of *OsAAP6* in all three kinds of transformants, OX(NYZ), ZpZc(NYZ) and RNAi(ZS97) (Supplementary Table 3). Transgenic plants showing higher levels of *OsAAP6* expression produced larger amounts of grain storage protein (Supplementary Table 3 and Table 1). Thus, our results confirm that the candidate gene (*OsAAP6*) is the *qPC1* QTL.

### Expression pattern of *OsAAP6* and subcellular localization

**Figure 2:**
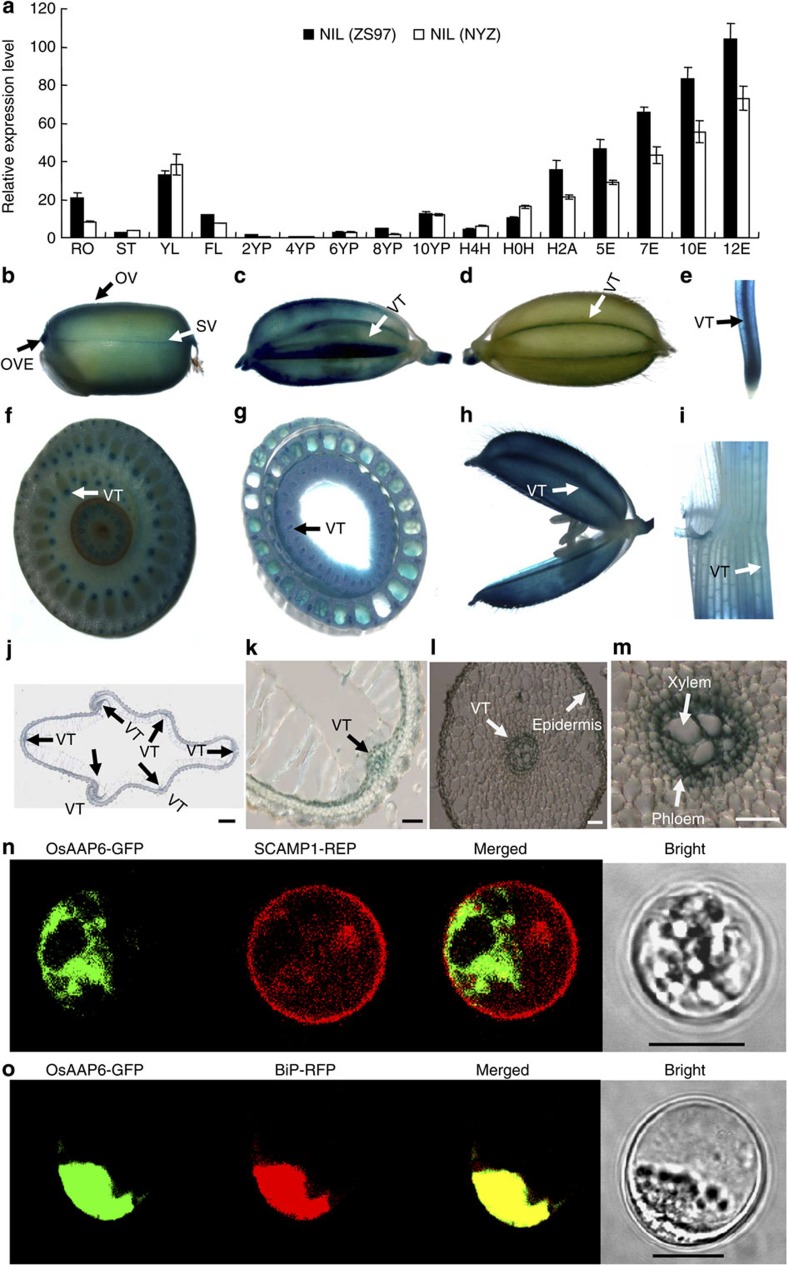
Expression patterns of *OsAAP6* and subcellular localization analysis. (**a**) The comparative expression pattern of *OsAAP6* was determined by quantitative reverse transcription–PCR. RO, ST and FL: root, stem and flag leaf at the heading stage; YL, young leaf; 2YP, 4YP, 6YP, 8YP and 10YP: young panicles 2, 4, 6, 8 and 10 cm in length; H4H and H0H: hulls at 4 and 0 days before heading; H2A, hull at 2 DAF; 5E, 7E, 10E and 12E: endosperms at 5, 7, 10 and 12 DAF. All data are based on three biological replications. Error bars, s.e.m. (**b**–**m**) Representative histochemical analysis of tissue expression of *GUS* transgene under control of the *OsAAP6* promoter from ZS97. (**b**) Endosperm 10 DAF. (**c**) Seed 10 DAF. (**d**) Seed 25 DAF. (**e**) Root. (**f**) Node. (**g**) Internode. (**h**) Hull at 2 days before flowering. (**i**) Pulvinus. (**j**,**k**) Hull cross sections. (**l**,**m**) Root cross sections. VT, vascular tissue; OVE, ovular vascular trace ends; OV, ovular vascular region; SV, lateral stylar vascular traces. Scale bars, 1 mm (**j**), 50 μm (**k**–**m**). All transgenic plants were from the T_1_ generation, and at least ten independent transformants were subjected to histochemical GUS assays. (**n**,**o**) Subcellular localization analysis of OsAAP6. (**n**) *OsAAP6*-GFP does not co-localize with SCAMP1-RFP, which is located in the *trans*-Golgi network and plasma membrane, as a control. (**o**) Co-localization of OsAAP6-GFP and BiP-RFP, which is located in the ER. Merged images of YFP and bright-field images are also shown. Scale bar, 10 μm.

Sixteen tissues from the NILs were assayed for temporal and spatial expression patterns of *OsAAP6* by quantitative reverse transcription–PCR (Fig. 2a). *OsAAP6* transcripts were detected in all examined tissues and were most abundant in the endosperms at 5–12 days after flowering (DAF) (Fig. 2a), the same timeframe as reported for transcripts of *OsAAP6* (ref. 23), and there were significantly higher transcript levels in the endosperms of NIL(ZS97) than in endosperm of NIL(NYZ) (Fig. 2a).

We next examined the expression of the β-glucuronidase (*GUS*) gene under control of the *OsAAP6* promoter from ZS97 in transgenic plants. All tissues exhibited GUS activity and the GUS signal was particularly strong in vascular tissues of the hull, root, pulvinus, internode, node, seed and endosperm at 10 and 25 DAF (Fig. 2b–m). GUS activity in the endosperm was more abundant in the ovular vascular trace, ovular vascular trace end and lateral stylar vascular traces than in other positions during grain filling (Fig. 2b). Histochemical GUS studies showed that *OsAAP6* was expressed mainly in the root rhizodermis and phloem (Fig. 2l,m). This feature of *OsAAP6* closely resembles *Arabidopsis* AAP family genes and *LHT1*, which are preferentially expressed in vascular tissues and the root rhizodermiss^24^,^25^,^26^,^27^.

To investigate the subcellular localization of OsAAP6 protein, we transiently co-expressed OsAAP6 fused to green fluorescent protein (GFP) with various subcellular markers in rice protoplasts^28^,^29^,^30^,^31^. Co-expression of OsAAP6-GFP and SCAMP1-RFP clearly showed that OsAAP6 was not localized at the plasma membrane or on the *trans*-Golgi network (Fig. 2n). However, OsAAP6-GFP and the endoplasmic reticulum (ER) marker BiP-RFP overlapped completely when co-expressed in rice protoplasts (Fig. 2o). Therefore, *OsAAP6* is localized on the ER, but not on the plasma membrane or in the *trans*-Golgi network.

### Genetic variation in the regulatory region of *OsAAP6*

**Figure 3:**
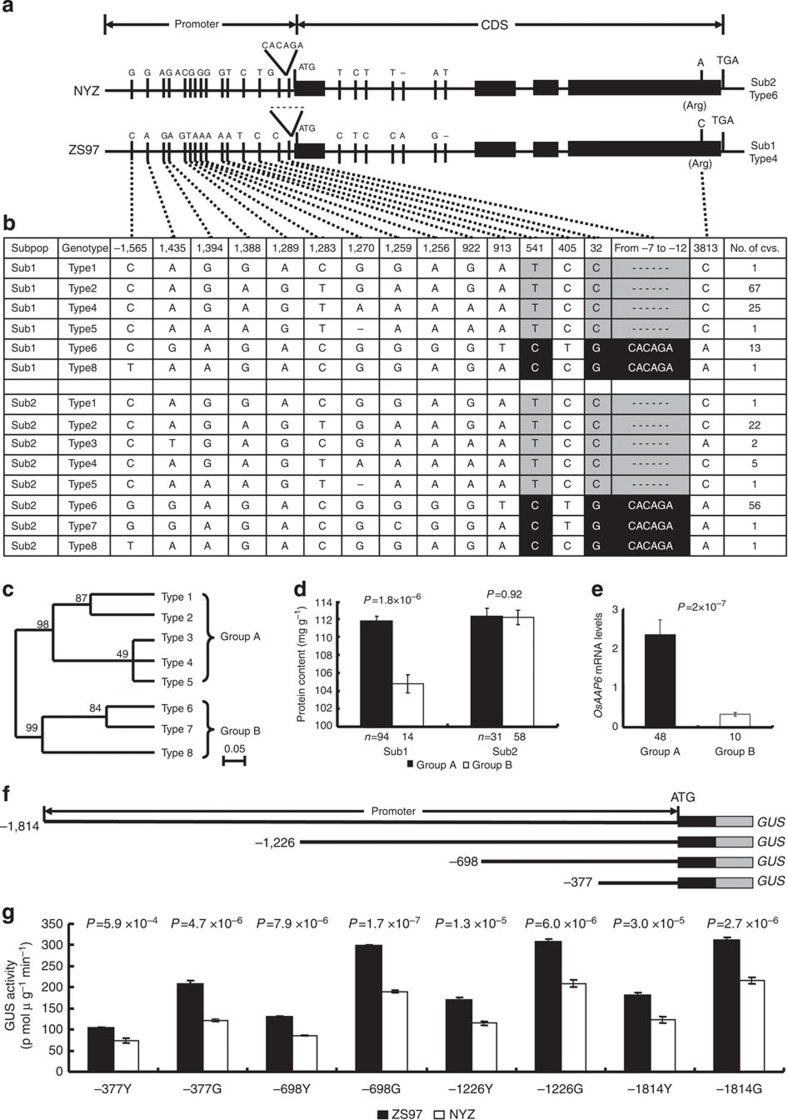
Natural variation of *OsAAP6* in a 197 accession rice mini-core collection and GUS activities in transgenic plants driven by eight promoter fragments and 5′-UTRs. (**a**) *OsAAP6* gene structure and natural variation between alleles from ZS97 and NYZ. (**b**) Natural variation of *OsAAP6* in 197 rice accessions of a mini-core collection compared with the NILs. (**c**) Cladogram of eight haplotypes. (**d**) Protein contents of brown rice in sub-populations A and B; raw data are provided in Supplementary Table 5; *n*, is the number of accessions. *P*-values were generated by two-tailed *t*-tests. Error bars, s.e.m. (**e**) *OsAAP6* transcript levels in the endosperms of Sub1 cultivars with class A and B at 5 DAF; the number of accessions analysed is shown below each bar. The *P*-value was generated by a two-tailed *t*-test. Error bars, s.e.m. (**f**) Diagrams for the four deletions of the *OsAAP6* promoter and 5′-UTR fused to the *GUS* gene. (**g**) Quantitative analysis of GUS activity in transgenic plants. Y and G indicate young panicles at 2 days before flowering and grains at 5 DAF, respectively. Data were from the transgenic lines planted in a randomized complete block design with three replications. *P*-values were produced by the Duncan test. Error bars denote s.e.m.

To identify the sequence variation between the ZS97 and NYZ alleles of *OsAAP6*, we compared genomic clones from both parents (Fig. 3a). There were eight nucleotide differences between the two varieties in the open reading frame: seven polymorphisms in the first intron and one synonymous mutation (at 3,813 bp) in the fourth exon (Fig. 3a). In addition, 15 polymorphisms between ZS97 and NYZ were revealed in the 5′-untranslated region (5′-UTR) and promoter region (~1.8 kb). These polymorphisms included a 6-bp indel and 14 upstream single-nucleotide polymorphisms (Fig. 3a). Several types of putative regulatory elements were identified within the polymorphic regions of the *OsAAP6* promoter and 5′-UTR, which harbours several *cis*-regulatory elements involved in transcriptional responses, such as a copper-response element, inr element, ARR1-binding element and other *cis*-elements (Supplementary Table 4).

We sequenced the *OsAAP6* 5′-UTRs and promoter regions (~1.8 kb), and the coding regions containing the synonymous mutation site, and measured GPC in 197 accessions of the rice mini-core collection originating from a wide geographic range across Asia (Supplementary Table 5). The collection comprised two subpopulations (Sub1 and Sub2) identified as previous population structure analyses^32^,^33^. Based on the nucleotide polymorphisms identified between the two parents (Fig. 3a), the sequences of the cultivated varieties were divided into eight haplotypes (Fig. 3b), placed into two groups (A and B) by phylogenetic analysis of the sequences. Five haplotypes were present in Group A (named types 1–5) and three in Group B (types 6–8) (Fig. 3c and Supplementary Table 5). Sub1 with 94 accessions in Group A and 14 accessions in Group B comprised mainly *indica* cultivars, and Sub2 with 31 accessions in Group A and 58 accessions in Group B were mainly *japonica* (Fig. 3b,c). Cultivars in Sub1 carrying Group A haplotypes tended to show higher *OsAAP6* expression levels in endosperms at 5 DAF and a higher GPC than those having the class B haplotypes (Fig. 3d,e). We also assayed the transcript abundance of *OsAAP6* in endosperms at 5 DAF (Fig. 3e) and calculated the correlation between *OsAAP6* expression levels and GPC in the *indica* group. The highly significant correlation (0.65, *P*<0.01) strongly suggests that expression levels for this gene might be the cause of natural variation in *indica* GPC. Furthermore, three common nucleotide polymorphisms upstream of the translation start site were identified between Groups A and B (Fig. 3b), and two of the common nucleotide changes (−7 to −12 bp, −32 bp) in the 5′-UTR were in three potential *cis*-regulatory elements (copper-responsive element, inr element and sulphur-responsive element) (Supplementary Table 4), which are targets for transcriptional activators and a regulator containing an SBP domain, and are involved in a broad range of responses^34^,^35^,^36^,^37^,^38^,^39^,^40^. Consequently, our results imply that the two common variations in the three potential *cis*-elements of the *OsAAP6* 5′-UTR seem to be associated with GPC diversity in the Sub1 population (mainly *indica* cultivars).

A series of 5′-end deletions of the *OsAAP6* promoter from ZS97 and NYZ were fused to the *GUS* gene (Fig. 3f), and their ability to drive the reporter gene was assessed relative to the full-length ZS97 promoter in transgenic plants. Compared with fragments of −377, −698, −1,226 and −1,814 bp from NYZ, quantitative analysis revealed that the GUS expression levels from ZS97 were significantly increased in young panicles 2 days before flowering and in grains at 5 DAF (Fig. 3g). However, compared with fragments of −698 bp in grains at 5 DAF, the GUS expression levels from the fragments of −377 bp from ZS97 and NYZ were markedly decreased (*P*<0.01), respectively (Fig. 3g). Thus, the minimal region (−698 to +1) of *OsAAP6* may play an important role in regulating the *OsAAP6* expression difference between the alleles from ZS97 and NYZ.

### Effects of *OsAAP6* on grain nutritional quality

To quantify the effects of *OsAAP6* on grain quality, we first assayed the quality traits of the NILs and transgenic plants of OX(NYZ), OX(ZH11), ZpZc(NYZ), RNAi(ZS97) and RNAi(ZH11) using data from field trials (Table 1). NIL(ZS97) and transgene-positive plants of OX(NYZ), OX(ZH11) and ZpZc(NYZ) had substantially increased GPC (grain storage proteins, including glutelins, prolamins, globulins and albumins) and amylose contents, coupled with markedly reduced starch content and gel consistency, whereas the reverse was true for RNAi(ZS97) and RNAi(ZH11) (Table 1). There were no significant differences between the NILs and transgenics in other agronomic traits (Supplementary Fig. 4 and Supplementary Table 6).

The total amino acid contents of grains were also determined for the NILs and the transgenic plants. Compared with the corresponding levels in NIL(NYZ) and transgene-negative OX(NYZ) plants, the levels of alanine, leucine, valine, proline, arginine, acidic amino acids and total content of amino acids were significantly increased in NIL(ZS97) and transgene-positive plants, whereas the reverse was true for RNAi (ZH97) (Supplementary Fig. 5). These results strongly suggest that *OsAAP6* enhances GPC by increasing grain storage protein (glutelins, prolamins, globulins and albumins) content and the total amount of amino acids, thus improving nutritional quality.

### Changes in amino acids of root uptake and distribution

**Figure 4:**
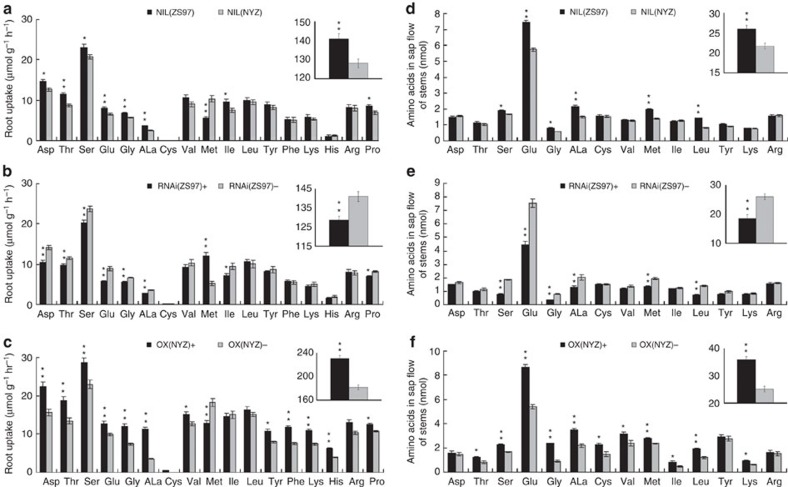
Amino acid uptake and sap flow assays. (**a**–**c**) The effect of *OsAAP6* on amino acid uptake by rice roots from NILs (**a**), RNAi (**b**) and OX(NYZ) (**c**). Plants were incubated in solutions containing a mixture of 20 amino acids, each at a concentration of 50 mM; rates of depletion of amino acids from this solution were determined. Root measurements were calculated using fresh weights. (**d**–**f**) Amino acid analysis of sap flow of stems from NILs (**d**), RNAi (**e**) and OX(NYZ) (**f**). Aliquots of 20 μl of stem sap were collected and assayed. Insert indicates the total content of amino acids. (+) and (–) indicate transgene-positive and negative T_2_ plants, respectively. Significant differences at *P=0.05 and **P=0.01, respectively. All data are based on three biological replications and significant differences are based on two-tailed *t*-tests. Error bars, s.e.m. Glutamine (Gln) and asparagine (Asn) were hydrolysed to glutamate (Glu) and aspartate (Asp) under acidic conditions; thus, the final content of Glu was exactly the sum of the Gln and Glu contents and the final content of Asp was exactly the sum of the Asn and Asp contents.

As *OsAAP6* was preferentially expressed in the vascular tissues and rhizodermis of roots, we examined the effects of *OsAAP6* on root uptake and distribution of various free amino acids. First, roots were submerged in a solution containing a mixture of 20 amino acids, and the depletion of each amino acid in this solution was measured 6 h after incubation. The study was not designed to establish actual rates of amino acid absorption by the plants, but rather to compare the NILs and transgenic plants with respect to this process. NIL(ZS97) and transgene-positive plants of OX(NYZ) had significantly increased threonine, serine, glycine, alanine, proline, acidic amino acids and total amino acids content, coupled with significantly reduced methionine, whereas the opposite was true for RNAi(ZS97) (Fig. 4a–c). The uptake data suggest that *OsAAP6* greatly enhanced root absorption of a range of amino acids and displayed substantially higher uptake rates of threonine, serine, glycine, alanine, proline and acidic amino acids.

Uptake (or synthesis) of amino acids mainly takes place in mature roots and leaves; they are then exported via the stems to supply the flowers and grains. Sap flow in stems was analysed to determine whether *OsAAP6* might play a role in this process. Serine, glycine, alanine, methionine, leucine, glutamate (or glutamine) and total amino acids levels were increased in NIL(ZS97) and transgene-positive plants of OX(NYZ), whereas lower levels were observed for RNAi(ZS97) (Fig. 4d–f).

Free amino acids in flag leaves and hulls were investigated to further determine whether *OsAAP6* had any effect on the distribution of free amino acids. NIL(ZS97) and transgene-positive plants of OX(NYZ) showed substantial decreases in serine, alanine, valine, methionine, tyrosine, acidic amino acids and total content of free amino acids in flag leaves (at 10 DAF), whereas the opposite was true for the RNAi(ZS97) (Supplementary Fig. 6a–c). However, in hulls many free amino acids and total content of free amino acids were significantly increased in NIL(ZS97) and transgene-positive plants of OX(NYZ) (at 10 DAF), whereas the reverse was true for the RNAi(ZS97) (Supplementary Fig. 6d–f). Our results therefore suggest that *OsAAP6* has effects on the distribution of various amino acids, at least in stems, flag leaves and hulls.

### Pleiotropic effects of *OsAAP6* on gene expression

**Figure 5:**
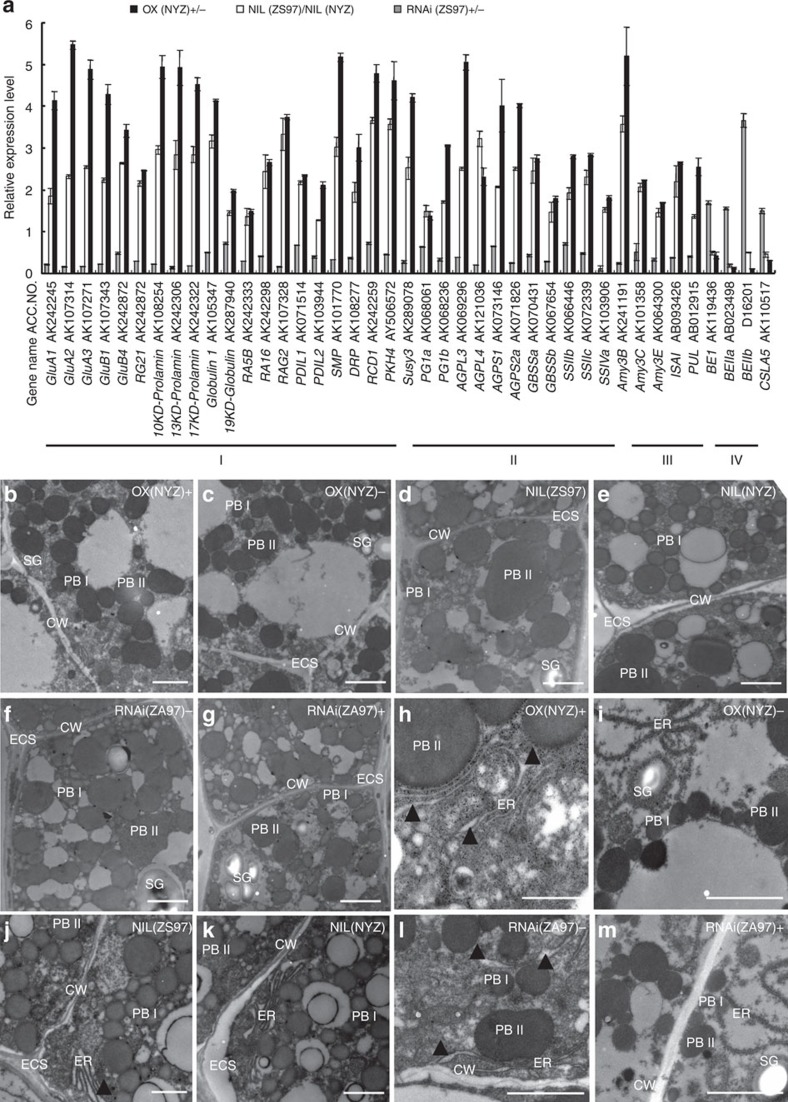
Expression levels of genes involved in production of storage starch and proteins and transmission electron microscopy. (**a**) Expression levels of key genes involved on synthesis and storage of endosperm components in transgenic plants (OX(NYZ) and RNAi(ZS97)) in T_2_ and NILs. Transgene-positive plants of *OsAAP6* and NIL (ZS97) are shown relative to transgene-negative plants and NIL (NYZ), respectively, set as 1. Data are based on three biological replications. All *P*-values, produced by two-tailed *t*-tests, were <0.01. Error bar shows s.d. The annotated names of the genes are shown in Supplementary Table 7. (**b**–**m**) Ultrastructures of cells in developing endosperm of NILs and transgenic plants at 10 DAF. PBI and PBII, protein bodies I and II; SG, starch granule; ER, endoplasmic reticulum; ECS, extracellular space; CW, cell wall. Scale bars, 2 μm.

To determine whether changes in the accumulation of grain storage proteins and starch were reflected by altered messenger RNA levels, we examined the expression of key genes involved in grain storage materials. The mRNA transcript levels of 41 genes, including 20 involved in grain protein biosynthesis (Fig. 5a, I), 17 related to starch metabolism (12 involved in amylose biosynthesis (Fig. 5a, II) and 5 related to starch degradation (Fig. 5a, III)) in endosperms at 10 DAF were considerably upregulated in OX(NYZ) and NIL(ZS97), in contrast to plants not carrying the transgene and NIL(NYZ). The reverse was true for 37 of the 41 genes in RNAi(ZS97) (Fig. 5a). Furthermore, the transcript levels of three genes involved in amylopectin biosynthesis were markedly downregulated in NIL(ZS97) and OX(NYZ) transgene-positive plants, relative to control plants, and the reverse occurred for these three genes in RNAi(ZS97) (Fig. 5a, IV). These results strongly suggest that *OsAAP6* has significant effects on the expression of a large portion of the genes participating in starch and storage protein biosynthesis in developing rice grains.

To test whether changes at the transcriptional level affected enzyme behaviour, the activities of five key enzymes involved in starch metabolism were measured. At 10 DAF, NIL(ZS97) and transgene-positive plants of OX(NYZ) had increased activities of ADP-glucose pyrophosphorylase (ADPG-Pase), granule-bound starch synthase (GBSS), soluble starch synthase (SSS) and amylase, coupled with reduced starch branching enzyme (SBE) activity in developing endosperms, whereas the opposite was true for RNAi(ZS97) (Supplementary Fig. 7a–o). These results are consistent with the expression of key genes involved in starch metabolism (Fig. 5a) and the contents of amylose and starch (Table 1).

### *OsAAP6* enlarges protein bodies

As *OsAAP6* greatly enhanced GPC in rice, transmission electron microscopy was employed to determine any effects on protein body (PB) formation. In developing endosperms (at 10 DAF), two types of PBs were readily discernible in ultra-thin sections; prolamin-containing PBI consistently shaped and surrounded by ER, and irregularly shaped glutelin/globulin-containing PBII showing uniform staining (Fig. 5b–m). In the developing endosperms of NIL(ZS97) and OX(NYZ), the mean section areas of PBI and PBII were all expanded, whereas the opposite was true for RNAi(ZS97) (Fig. 5b–g and Supplementary Fig. 8). Interestingly, the ER cisternal space was often dilated in transgene-positive plants of OX(NYZ) and NIL(ZS97), as well as transgene-negative plants of RNAi(ZS97) (Fig. 5h–m, black arrowheads). These results indicate that *OsAAP6* has probable effects on the formation of PBs through enlargement.

## Discussion

Rice grain quality is a complex character, and the demand for high-quality cereals as a source of protein has become increasingly evident^4^,^41^. Although numerous QTLs for GPC have been detected^17^,^18^,^19^,^20^,^21^,^22^, no QTL controlling GPC underlying natural variation in rice has been cloned, and knowledge of the regulatory mechanism determining GPC was still lacking. In this study, we cloned a major QTL *OsAAP6* that controls GPC by regulating the synthesis and accumulation of grain storage proteins and starch. Our results revealed that *OsAAP6* functions as a positive regulator of GPC and greatly enhances the total amount of amino acids. Therefore, increasing the expression of *OsAAP6* in low GPC rice varieties can increase GPC as well as the total amount of amino acids, and thus improve grain nutritional quality.

Amino acid transporters/permeases are key regulators of plant metabolism, growth and development^24^,^25^. The *AAP* family consists of eight members (*AtAAP1*–*8*) in *Arabidopsis* and 19 members (*OsAAP1*–*19*) in rice^23^. Our study showed that *qPC1* in rice corresponds to *OsAAP6*, which is highly homologous to *AtAAP* family genes (*AtAAP1–8*) in *Arabidopsis* and other putative genes in the staple cereals (Supplementary Fig. 9). Furthermore, comparison of the locations of QTLs controlling GPC with the 19 genes (*OsAAP1–19*) in rice indicated that *OsAAP5* and *OsAAP8* are also located on the long arm of chromosome 1, whereas the other 16 *OsAAPs* are not closely associated with GPC QTLs^22^,^23^. Interestingly, *OsAAP5* and *OsAAP8* are barely expressed in seed^23^.

In plants, *AAPs* appear to be involved in many physiological processes^23^,^24^,^25^,^42^,^43^,^44^,^45^. The amount of protein was significantly decreased in desiccated seeds from *AtAAP2* T-DNA insertion lines^44^. However, overexpression of *VfAAP1* in both pea and *Vicia narbonensis* resulted in increased seed protein content^46^. Although the roles of many *AtAAPs* were revealed in *Arabidopsis*, no *OsAAP* was functionally characterized in rice^23^. Here we isolated and characterized *OsAAP6*, and our results suggest that *OsAAP6* has pleiotropic effects on grain storage materials (Table 1) and the expression of a large portion of the genes in developing rice grains (Fig. 5a).

In *Arabidopsis*, expression of *AtAAPs* is regulated by a complex array of *cis*-elements and transcription factors^24^,^25^. Sulphur-responsive element, copper-response element and inr element are targets for transcriptional activators/regulators and are involved in a broad range of responses^34^,^35^,^36^,^37^,^38^,^39^,^40^, including transcriptional activation/repression^34^,^35^,^36^,^37^, hormone signalling and fruit ripening^38^,^39^. Our results showed that the two common variations (−7 to −12 bp, −32 bp) in the three potential *cis*-regulatory elements seem to be associated with GPC diversity in *indica* cultivars (Fig. 3b–e and Supplementary Table 4), whereas there was no significant difference in GPC between Groups A and B in the *japonica* group using phenotypic data from years of 2010 and 2011 in Wuhan (Fig. 3d and Supplementary Table 5). This phenomenon may be due to the different genetic backgrounds between *indica* and *japonica*, and one gene is not possible to explain all the phenotype of GPC, which is controlled by many QTLs^17^,^18^,^19^,^20^,^21^,^22^. Further investigation indicated that these potential *cis*-elements may play important roles in regulating the *OsAAP6* expression difference between ZS97 and NYZ (Fig. 3f,g). Our results suggest that *OsAAP6* expression may be regulated by the copper-responsive element, inr element and sulphur-responsive element separately or by joint interaction with transcription regulators. Although detailed studies of the cause of the GPC diversity need to be addressed, our results provide important information for identifying functional variations of *OsAAP6*.

Based on our results, we propose a model to explain the role of *OsAAP6* in regulation of grain storage materials (Supplementary Fig. 10). We suggest that *OsAAP6* functions as a positive regulator of GPC (Table 1, Supplementary Table 3 and Fig. 3). Increasing the expression level of *OsAAP6* results in upregulation of key genes for storage proteins (Fig. 5a) and enhancement of root absorption of a range of amino acids (Fig. 4a–c), which may be distributed through the vascular tissues to the grains for biosynthesis and deposition of grain proteins (Figs 2b–m and 4d–f, and Supplementary Fig. 6a–f), finally leading to larger PBs (Fig. 5b–g and Supplementary Fig. 8) and higher GPC (Table 1). *OsAAP6* may indirectly effect the key genes for starch (amylose and amylopectin) biosynthesis (Fig. 5a, Supplementary Fig. 7a–o and Table 1).

Grain yield and quality are the two most important traits in rice research and breeding^41^, while GPC and amino acid composition are key factors determining the nutritional quality of cereal grains^6^. Breeding cereals with high protein while maintaining yield has been difficult^2^, because GPC is inherited as a typical polygenic trait^1^,^17^,^18^,^19^,^20^,^21^ that is sensitive to environmental factors^17^. Grain storage proteins are encoded by complex multigene families^5^,^10^ and high GPC has negative effects on other traits^2^. Phylogenetic analysis of *OsAAP6* revealed that it is highly homologous to the other putative genes in maize, sorghum, barley and wheat (Supplementary Fig. 9). Interestingly, *OsAAP6* controlled GPC with no effect on plant morphology, flowering time or grain yield (Supplementary Table 6 and Supplementary Fig. 4), suggesting that GPC and nutritional quality could be improved without reduction in grain yield. Thus, map-based cloning and characterization of *OsAAP6* have paved the way for genetic improvement of GPC and grain nutritional quality in rice and, potentially, other staple cereals.

## Methods

### Primers

Primers used in this study are listed in Supplementary Tables 7 and 8.

### Map-based cloning of the *qPC1* QTL

Two BC_3_F_2_ populations consisting of 6,000 (population 1) and 4,008 (population 2) individuals were planted in the winter of 2008 on Hainan Island and in the summer of 2009 in an experimental field at Huazhong Agricultural University, respectively. To fine-map the *qPC1* locus, we developed new molecular markers (including SSR and InDel markers) from the sequences of *qPC1*-flanking regions of Nipponbare and used them for genotyping. All of the recombinant plant genotypes at the *qPC1* locus were determined by progeny testing in Wuhan.

### Natural variation of *OsAAP6* in a rice mini-core collection

We sequenced the genomic region of *OsAAP6* in the NILs and 197 accessions of a rice mini-core collection, which included 101 cultivars of *O. sativa* ssp. *indica* and 96 cultivars of *O. sativa* spp. *japonica* originating from a wide geographic range across Asia. The population structure of the mini-core collection of 197 accessions was investigated by the model-based method implemented in STRUCTURE^32^,^33^. Total protein content of the grain was measured with an XDS Near-Infrared Rapid Content Analyzer (NIR, FOSS) by means of near-infrared reflectance spectroscopy^47^. Briefly, a Foss XDS spectrometer was used to obtain near-infrared spectral data between 400 and 2,500 nm at 2-nm intervals. The relationship between NIR spectral patterns of each sample and its GPC was examined, and data analysis was conducted using the WinISI software. The best predicted equation for GPC was selected on the basis of minimizing the s.e. of cross-validation and increasing the coefficient of determination, and the accuracy of model was evaluated on the validation subset, using coefficient of determination of the cross validation, slope and residual predictive value.

### Plant growth conditions and trait measurement

Rice plants were examined under field conditions at the experimental stations of Huazhong Agricultural University in Wuhan and Hainan. The planting density was 16.5 cm between plants within rows and 26 cm between rows. Fields were managed according to local agricultural practices. Harvested rice grains were air-dried and stored at room temperature for at least 3 months before testing. Fully filled grains were used for measuring grain quality and yield traits. Milled rice was ground to flour for measurement of amylose content, starch content and gel consistency^48^. Glutelins, prolamins, globulins and albumins were extracted sequentially from seeds using three solvents, and quantitative analyses of the proteins were executed by TECAN Infinite M200 (refs 15, 49). Grain weights determined on samples of 200 grains were converted to 1,000-grain weights.

### RNA preparation and reverse transcription

Total RNA was extracted from various plant tissues with an RNA extraction kit and TRIzol reagent (Invitrogen). First-strand cDNA was synthesized with 2 μg of RNA and 200 U of M-MLV reverse transcriptase (Promega) in a volume of 20 μl.

### Constructs and transformation

To prepare the complementation construct (ZpZc), we fused the 1.8-kb promoter fragment and 5′-UTR of *OsAAP6* from ZS97 with the coding region from ZS97; the *OsAAP6* promoter, 5′-UTR and coding region were obtained by PCR from ZS97 and confirmed by sequencing. The coding region from ZS97 was first inserted into the plant binary vector pCAMBIA1301, and then the promoter fragment and 5′-UTR was fused with it. To prepare the overexpression (OX) construct, we inserted the *OsAAP6* coding region of ZS97 into the plant binary vector pCAMBIA1301S under control of the CaMV 35S promoter. To generate the RNAi construct, we cloned a PCR fragment containing 580 bp from the fourth exon of *OsAAP6* (nucleotides 3,364–3,943) into the dspCAMBIA1301 vector in both the sense and the antisense orientations. The OX construct was introduced into the rice cultivars NYZ (OX(NYZ)) and ZH11 (OX(ZH11)), and ZpZc was introduced into NYZ (ZpZc(NYZ)) by means of *Agrobacterium*-mediated transformation. We introduced the RNAi construct by means of *Agrobacterium*-mediated transformation into ZS97 and ZH11 (RNAi(ZS97) and RNAi(ZH11), respectively) by means of a previously described procedure^50^,^51^, with minor modifications (each subculture was conducted on a subculture medium at 25 °C in the dark for 18 days). The 5′ deletions of the *OsAAP6* promoter from ZS97 and NYZ at positions −1,814, −1,226, −698 and −377 were generated by PCR amplification and inserted into expression vector pDX2181 (ref. 52). The resulting constructs (designated as −1,814, −1,226, −698 and −377 from ZS97 and NYZ4, respectively) were introduced into *Agrobacterium tumefaciens* strain EHA105 and transferred into ZH11 by *Agrobacterium-*mediated transformation^50^.

### Quantitative real-time PCR

Quantitative real-time PCR was carried out in a total volume of 25 μl containing 2 μl of the reverse-transcribed product, 0.2 mM of gene-specific primers, 12.5 μl of SYBR Premix EX Taq and 0.5 μl of Rox Reference Dye II (Takara) on an ABI 7500 real-time PCR system, according to the manufacturer’s instructions. Measurements were obtained by means of the relative quantification method^53^. The rice *Actin1* gene was used as the internal control. All expression level data obtained by quantitative real-time PCR are based on at least two biological samples on which three replications of the technique were conducted.

### Histochemical and fluorometric GUS assays

GUS activity in the various tissues of transgenic-positive plants was localized histochemically^52^, and photographs were taken under a dissecting microscope. Tissues of transgenic plants were incubated overnight in GUS staining solution at 37 °C, dehydrated and embedded in paraffin (Sigma). The tissues were sliced into 10-μm sections with a microtome and fixed to microscope slides and examined under a fluorescence microscope. For each construct, at least ten independent transformants were subjected to histochemical GUS assays. The protein concentration in the supernatant was quantified by means of the Bradford assay^49^. GUS protein concentration in the supernatant was determined fluorometrically with an INFINITE 200 photometer (Tecan Austria Gmbh). For each construct, 15 GUS staining-positive transformants were used for quantitative analysis of GUS activity. Three biological replicates per construct were analysed by fluorometric assays and averages were calculated.

### Assays of enzyme activities

Endosperms of 50 dehulled grains were separated from the embryos and pericarps, and homogenized in a pre-cooled mortar containing 5 ml frozen extraction medium. The homogenate was centrifuged at 12,000 *g* for 20 min at 4 °C, the extracted solution was used for measuring enzyme activities and the precipitate was used for measuring the granule-bound starch synthase activity, which was assayed by TECAN Infinite M200 (refs 54, 55). The extracted solution was assayed for the activities of ADPG-Pase, SSS, amylase and SBE^56^. The activities of ADPG-Pase and SSS were defined as the NADPH amount by measuring increases in absorbance at 340 nm, and SBE activity was defined as the decrease in amount of 1% KI-I_2_ at 540 nm. Three replications were conducted for every experiment.

### Subcellular localization of the OsAAP6 protein

The full-length *OsAAP6* cDNA was amplified by PCR using cDNA obtained from reverse transcription. The amplified *OsAAP6* cDNA fragment was inserted into a premade GFP vector in pM999 via *Sac*I and *Xba*I restriction enzyme sites to generate OsAAP6-GFP, and PEG-mediated transfections were carried out^57^. Well-established fluorescent protein markers BiP-RFP for the ER^28^ and SCAMP1-RFP for the *trans*-Golgi network and plasma membrane^29^,^30^,^31^ were used. Protoplasts were observed using a confocal laser scanning microscope and visualized by a Leica Microsystem LAS AF. All fluorescence experiments were independently repeated at least three times.

### Transmission electron microscopy analyses

Transverse sections (1-mm thick) of developing seeds were fixed for over 12 h in 2.5% glutaraldehyde buffered with 0.2 M phosphate buffer (pH 7.2). The sections were treated and sectioned with an ultramicrotome (Power Tome-XL, RMC). Microscopic observation was performed using a transmission electron microscope (H-7650, Hitachi). The area of PBs in each sample was determined using ImageJ software (NIH).

### Amino acid analysis

NILs and transgenic plants were grown in a rice culture medium^50^ under sterile conditions and placed in a climate chamber with a 14-h light/10-h darkness and 25 °C/18 °C (day/night) regime. After 20 days of growth, 3 plants of each plant type were selected for the uptake experiment, which was carried out on a shaking table in the climate chamber. The uptake solution was identical to the rice culture solution used during the plant cultivation, except that all N sources were replaced with a 20-amino-acid mixture composed of equal amounts of 50 mM solutions for each. Root uptake rates were determined from the decline in concentration of each amino acid in the solution during the 6 h incubation. The amino acid concentrations were determined using an automatic amino acid analyser of L-8800 (ref. 58).

Amino acid contents in the stem sap flow were obtained 10 DAF. Plants were cut 10 cm above the roots. Aliquots of 20 μl sap from the remaining stem bases were collected for 30 min after discarding the first drops, then lyophilized and re-suspended in 480 μl 0.02 M HCL and analysed by an L-8800 automatic amino acid analyser^58^.

Free amino acids in flag leaves and hulls, also at 10 DAF, were separately extracted with 40 ml of 80% ethanol. Aliquots (1 ml) of each sample were evaporated and the residues were re-dissolved in 1 ml of 0.02 M HCl and analysed with an L-8800 automatic amino acid analyser^58^.

## Author contributions

B.P. conducted most of the experiments, including fine mapping, gene cloning, expression analysis, genetic transformation, quality traits analysis, rice germplasm analysis, histochemical assays, quantitative analyses of GUS activity, promoter analysis, enzyme activities analyses and other functional analyses. L.J.L. constructed the segregating backcross populations; M.Z. and L.W. conducted the QTL primary mapping analysis. H.K. developed the NILs and participated in part of fine mapping. Y.L. carried out part of the expression analysis; G.W. and X.L. provided rice germplasm samples; W.X. and W.Y. carried out part of nucleotide diversity analysis; J.C. participated in part of the quality traits analyses. L.S., G.G., Q.Z., J.X., C.X. and X.L. participated in the promoter sequencing and rice germplasm analysis. Y.P. and L.L. participated in part of the enzyme activities analyses. Y.H. designed and supervised the study. Y.H. and B.P. analysed the data and wrote the paper.

## Additional information

**How to cite this article:** Peng, B. *et al.* *OsAAP6* functions as an important regulator of grain protein content and nutritional quality in rice. *Nat. Commun.* 5:4847 doi: 10.1038/ncomms5847 (2014).

**Accession codes:** Genomic DNA of *OsAAP6* in Zhenshan 97 has been deposited in GenBank/EMBL/DDBJ under the accession code KM213629. Genomic DNA of *OsAAP6* in Nanyangzhan has been deposited in GenBank/EMBL/DDBJ under the accession code KM213630.

## Supplementary Material

Supplementary Information Supplementary Figures 1-10 and Supplementary Tables 1-8

## Figures and Tables

**Table 1:** Grain quality traits in NILs and transgenic plants in a 2-year field trial.

**Genotype**	***n***	Protein content* **(mg** **g**^**−1**^**)**	**Protein content**† **(mg** **g**^**−1**^**)**	**Glutelins content**† **(mg** **g**^**−1**^**)**	**Prolamins content**† **(mg** **g**^**−1**^**)**	**Globulins and albumins content**† **(mg** **g**^**−1**^**)**	Amylose content* **(%)**	Starch Content* **(%)**	Gel consistency* **(mm)**
NIL(NYZ)	18	95.1±0.3	109.9±0.6	86.7±0.9	10.6±0.1	5.2±0.1	24.5±0.5	93.3±0.6	33.6±0.7
NIL(ZS97)	18	105.2±1.0	121.6±0.4	93.0±0.6	12.3±0.2	6.3±0.1	27.5±0.6	90.8±0.7	27.5±0.4
*P*‡		3.4 × 10^−3^	1.8 × 10^−5^	2.2 × 10^−3^	3.6 × 10^−4^	1.5 × 10^−3^	2.8 × 10^−3^	2.9 × 10^−3^	8.0 × 10^−3^
OX(NYZ)−	22	92.7±0.2	108.2±0.6	83.0±1.1	8.8±0.2	5.7±0.1	15.2±0.4	85.1±0.5	52.4±0.9
OX(NYZ)+	22	106.2±0.4	124.7±0.5	95.8±1.2	10.6±0.2	8.3±0.2	21.3±0.5	80.8±0.4	32.5±0.4
*P*‡		7.5 × 10^−6^	3.7 × 10^−6^	2.1 × 10^−4^	1.5 × 10^−4^	1.2 × 10^−4^	8.3 × 10^−5^	2.5 × 10^−4^	4.8 × 10^−5^
OX(ZH11)−	15	95.5±0.2	106.8±0.6	79.1±0.5	11.3±0.2	6.4±0.1	17.7±0.4	89.4±0.9	33.6±0.6
OX(ZH11)+	15	107.1±0.3	118.4±0.4	87.0±0.8	13.2±0.2	8.0±0.1	21.3±0.5	85.9±0.6	24.4±0.3
*P*‡		9.3 × 10^−7^	1.2 × 10^−5^	7.3 × 10^−4^	3.1 × 10^−4^	1.7 × 10^−4^	8.9 × 10^−3^	4.6 × 10^−3^	1.5 × 10^−4^
ZpZc(NYZ)−	24	92.6±1.6	108.7±0.4	84.1±0.9	8.9±0.3	5.8±0.1	15.2±0.4	84.8±0.4	52.3±0.7
ZpZc(NYZ)+	24	102.1±0.6	119.2±0.5	89.5±1.1	9.9±0.2	7.0±0.1	17.0±0.5	82.6±0.2	46.9±0.7
*P*‡		1.7 × 10^−3^	7.9 × 10^−6^	7.6 × 10^−4^	6.0 × 10^−3^	1.0 × 10^−4^	1.6 × 10^−4^	1.5 × 10^−3^	5.0 × 10^−6^
RNAi(ZS97)−	18	105.6±1.5	121.5±0.5	93.1±0.6	12.2±0.3	6.2±0.1	27.2±0.5	90.8±0.7	27.5±0.6
RNAi(ZS97)+	18	96.3±0.5	105.4±0.6	85.2±0.5	8.2±0.2	4.1±0.2	24.1±0.6	94.0±0.8	29.9±0.6
*P*‡		1.9 × 10^−3^	6.1 × 10^−4^	1.1 × 10^−3^	1.7 × 10^−4^	5.6 × 10^−5^	2.2 × 10^−3^	6.5 × 10^−3^	7.0 × 10^−3^
RNAi(ZH11)−	16	96.7±0.4	107.0±0.4	79.1±0.5	11.3±0.2	6.3±0.1	17.9±0.4	89.0±0.5	33.6±0.6
RNAi(ZH11)+	16	90.6±0.6	100.1±0.6	75.8±0.5	8.3±0.2	4.6±0.0	11.7±0.3	93.9±0.5	35.3±0.6
*P*‡		1.2 × 10^−4^	3.2 × 10^−5^	1.5 × 10^−3^	7.0 × 10^−5^	3.3 × 10^−5^	1.9 × 10^−5^	2.4 × 10^−4^	2.3 × 10^−2^

NIL, near-isogenic line; *n*, number of plants with the same genotype.

Data of all the materials are based on field trials using randomized block designs with three replications. All data are means±s.e.m. (+) and (−), transgene-positive and negative plants, respectively, and their data are the averages of 2 years. At least 300 grains of each plant were measured for all grain quality traits.

^*^Milled rice was used to determine protein content, starch content, amylose content and gel consistency.

^†^Brown rice was used to determine the protein content.

^‡^*P*-values are produced by Duncan’s test.
