# Supplementary material for: OsAAP6 functions as an important regulator of grain protein content and nutritional quality in rice
Source: Nat Commun. 2014 Sep 11;5:4847. doi: 10.1038/ncomms5847 (PMC4175581; doi:10.1038/ncomms5847)
Supplement: Supplementary Information — Supplementary Figures 1-10 and Supplementary Tables 1-8 [file ncomms5847-s1.pdf]

## Supplementary Figures

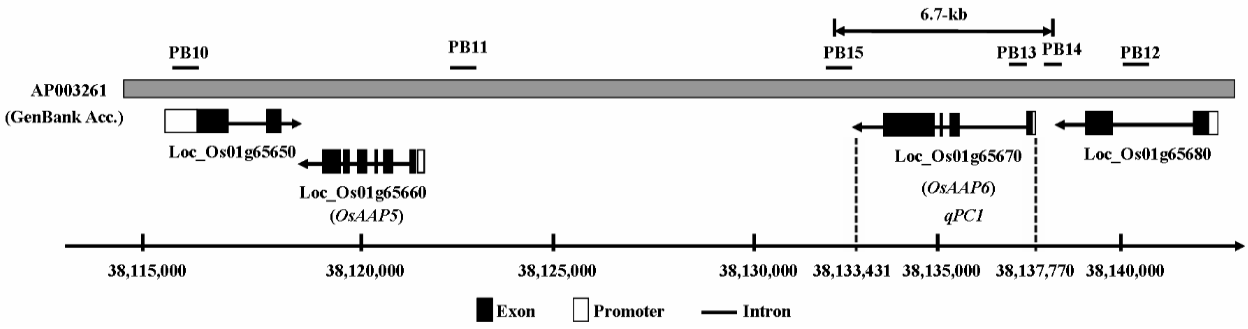


**Supplementary Figure 1 | Physical genomic map of the *qPC1* locus on rice chromosome 1 corresponding to Nipponbare, with marker and putative gene positions.**

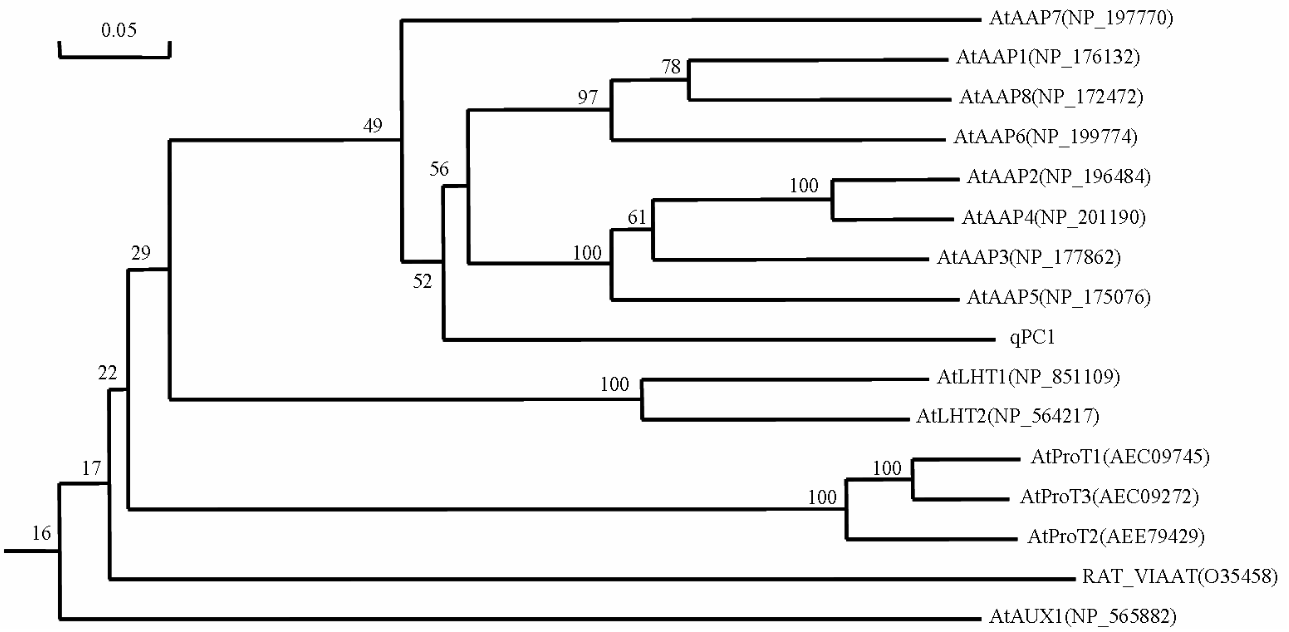


**Supplementary Figure 2 | Phylogenetic comparison of qPC1 protein with other amino acid transporters.** The phylogenetic tree of qPC1 protein and other transporters was generated by MEGA 4.0.

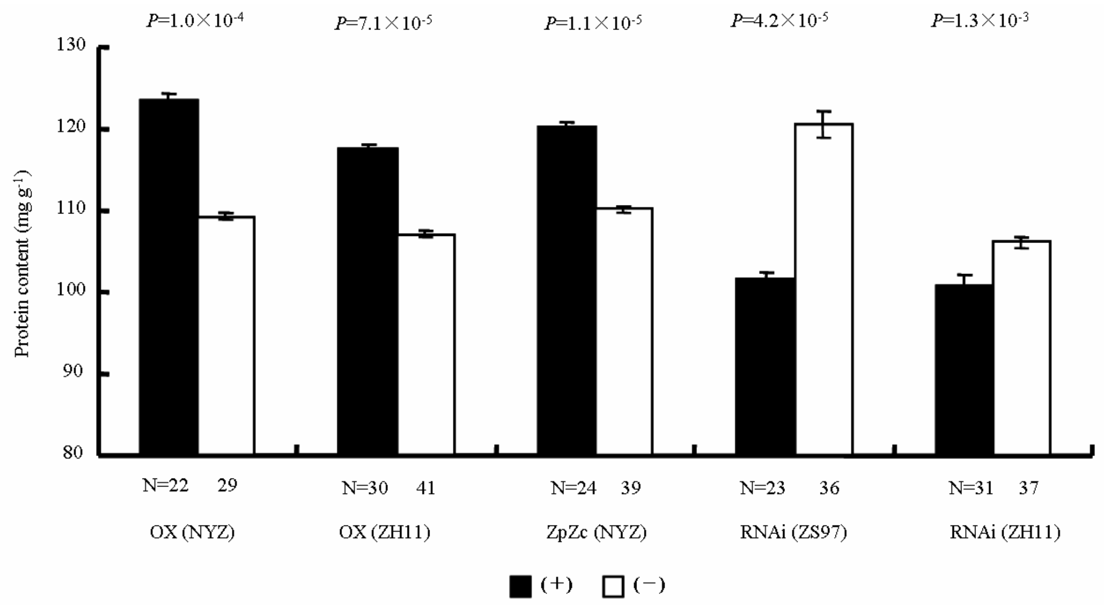


**Supplementary Figure 3 | Performance of transgene-positive and transgene-negative segregants in $T_0$ generation plants in the field trial.** (+) and (–) indicate transgene-positive and negative $T_0$ plants, respectively; N, number of plants; at least 300 grains of each plant were measured for brown rice protein content. *P* values were produced by two-tailed *t*-tests of two genotypic classes in the $T_0$ generation. Data are given as means ± s.e.m.

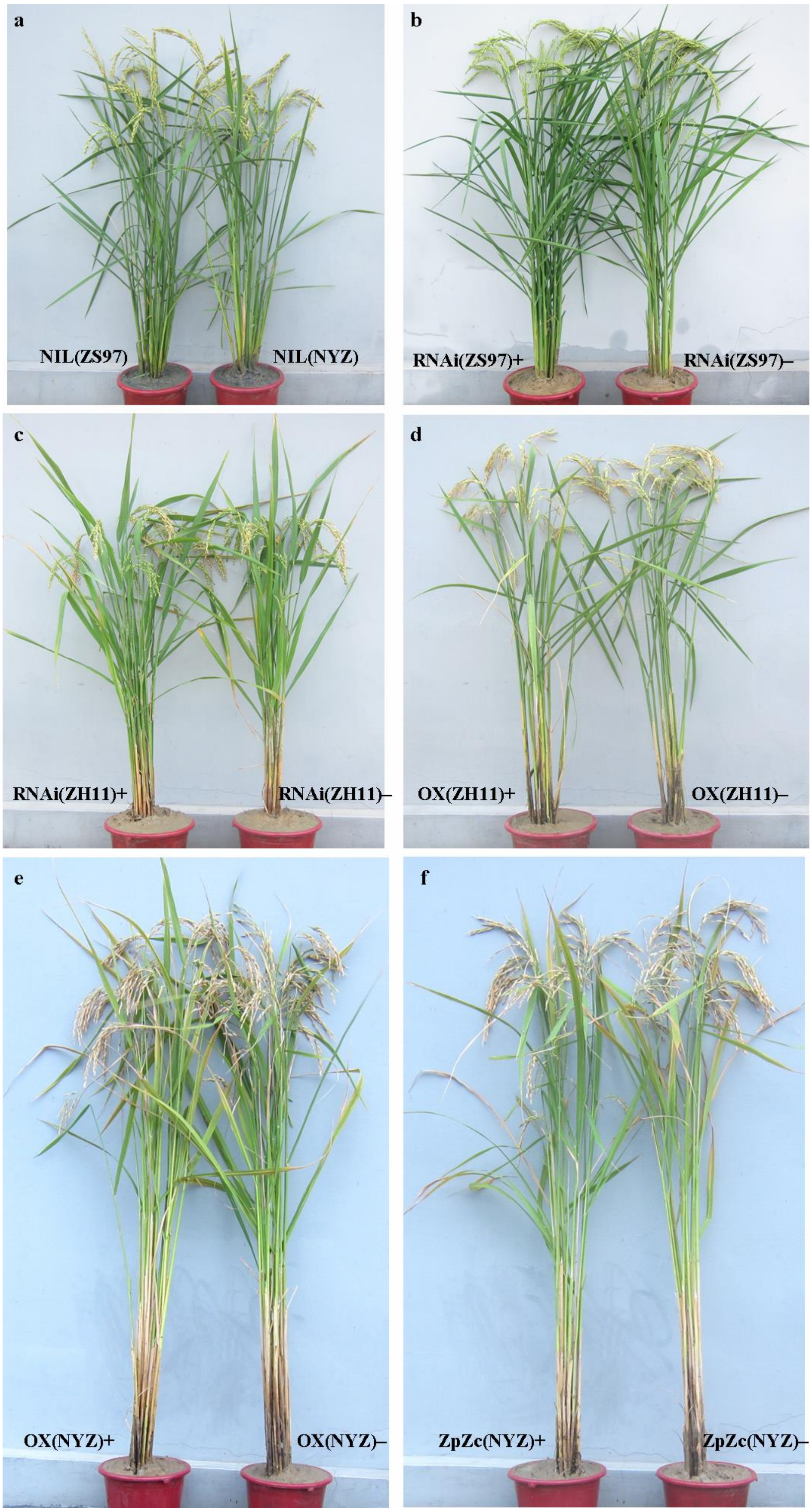


**Supplementary Figure 4 | Comparison of plant architectures of NILs, transgene-positive plants (+) and negative counterparts (–) in $T_2$.**

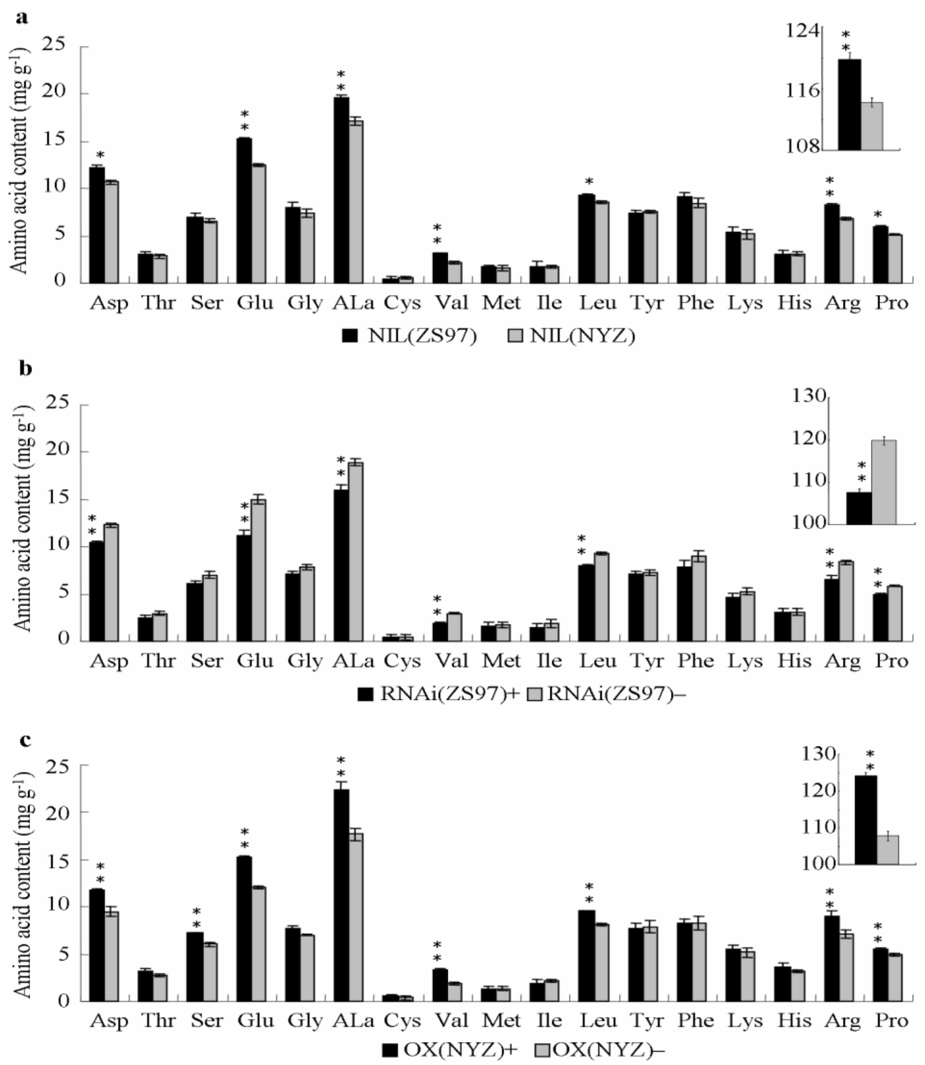


**Supplementary Figure 5 | Effects of *OsAAP6* on amino acids contents of the grain of NILs (a), RNAi (b) and OX(NYZ) (c).** Inserts indicate the total amino acids content. (+) and (–) indicate transgene-positive and negative $T_2$ plants, respectively. Data for all materials are based on a field experiment using a randomized complete block design with at least three replications; each replication comprised at least 15 plants with the same genotype and with at least 300 grains per plant; and all data are based on three biological replications, error bars, s.e.m. Significant differences at $*P = 0.05$ and $**P = 0.01$. Significant differences are based on two-tailed *t*-test. Glutamine (Gln) and asparagine (Asn) were hydrolyzed to glutamate (Glu) and

aspartate (Asp) under acidic conditions so the final content of Glu was the sum of the Gln and Glu contents and the final content of Asp was the sum of the Asp and Asn contents.

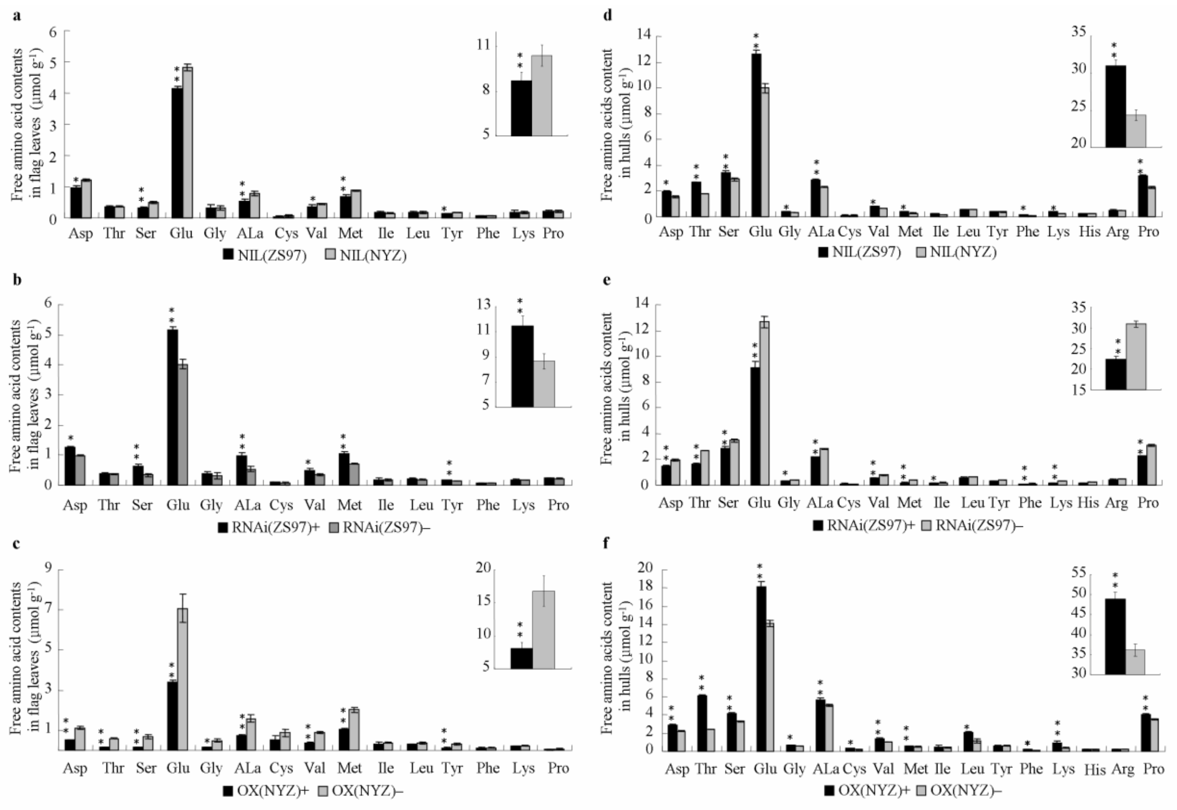


**Supplementary Figure 6 | Free amino acid composition and contents in flag leaves and hulls at 10 DAF.** (**a–c**) Free amino acid composition and content in flag leaves from NILs (**a**), RNAi (**b**) and OX(NYZ) (**c**). (**d–f**) Free amino acid composition and content in hulls from NILs (**d**), RNAi (**e**) and OX(NYZ) (**f**). Inserts indicate the total contents of free amino acids. (+) and (–) indicate transgene-positive and negative $T_2$ plants, respectively. The amino acid contents were measured by the fresh weights of flag leaves and hulls. All data are based on three biological replications, error bars, s.e.m. Significant differences $^*P = 0.05$ and $^{**}P = 0.01$. Significant differences are based on two-tailed *t*-test. Glutamine (Gln) and asparagine (Asn) were hydrolyzed to glutamate (Glu) and aspartate (Asp) under acidic conditions so the final content of Glu was the sum of the Gln and Glu contents, and the final content of Asp was the sum of the Asp and Asn contents.

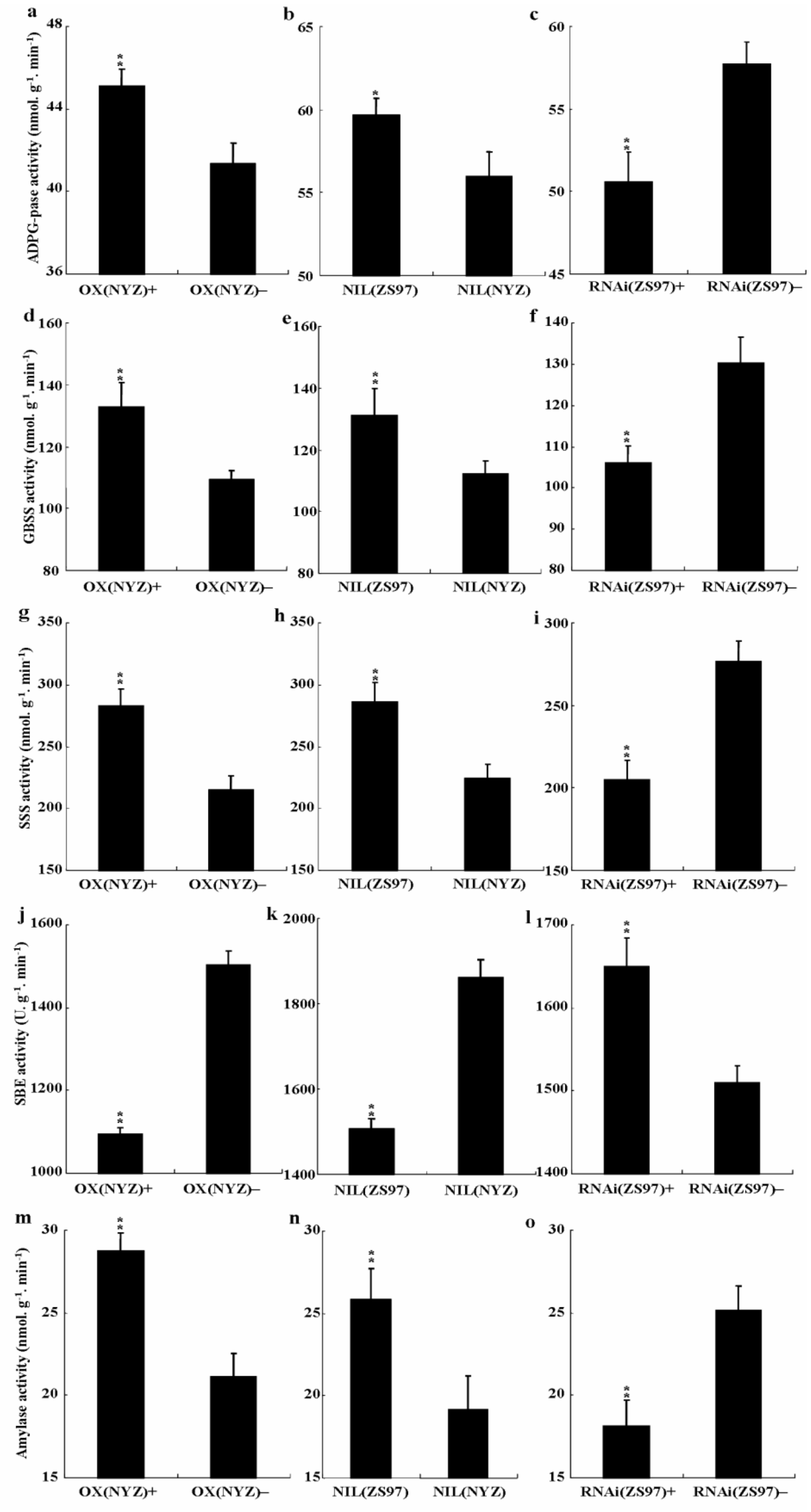


**Supplementary Figure 7 | changes on key enzyme activities in developing endosperms at 10 days after fertilization.** Activity changes in developing

endosperms of OX(NYZ), NILs and RNAi(ZS97) are shown for five key enzymes, ADPG-pase activity (**a–c**)**,** GBSS (**d–f**), SSS (**g–i**), SBE (**j–l**), and amylase (**m–o**). (+) and (–) indicate transgene-positive and negative $T_2$ plants, respectively. Error bars, s.e.m.; all data are based on three biological replications. Significant differences **P* = 0.05 and ***P* = 0.01. Significant differences are based on two-tailed *t*-test.

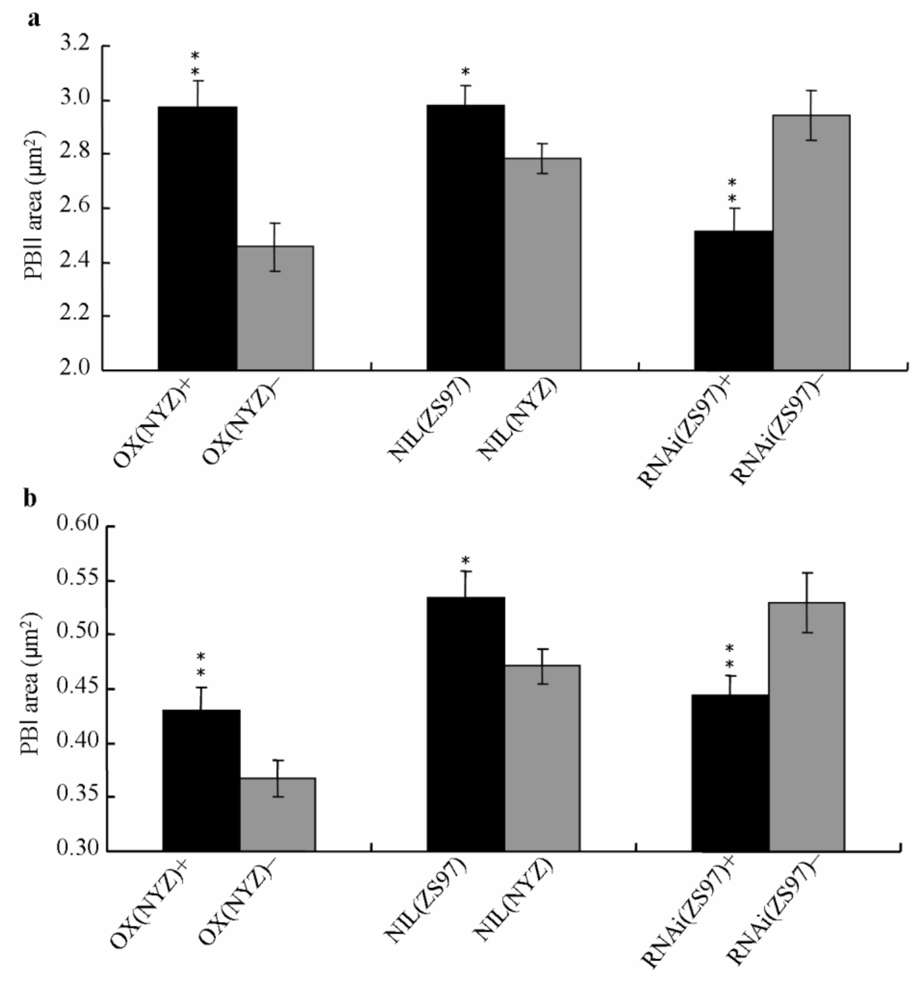


**Supplementary Figure 8 | Comparison of mean sectional areas of the two types of PB in endosperms from NILs and transgenic plants.** (**a**–**b**) Mean section areas of PBII (**a**) and PBI (**b**). (+) and (–) indicate transgene-positive and negative $T_2$ plants, respectively. Error bars, s.e.m. Significant differences at **P* = 0.05 and ***P* = 0.01. Significant differences are based on two-tailed *t*-test.

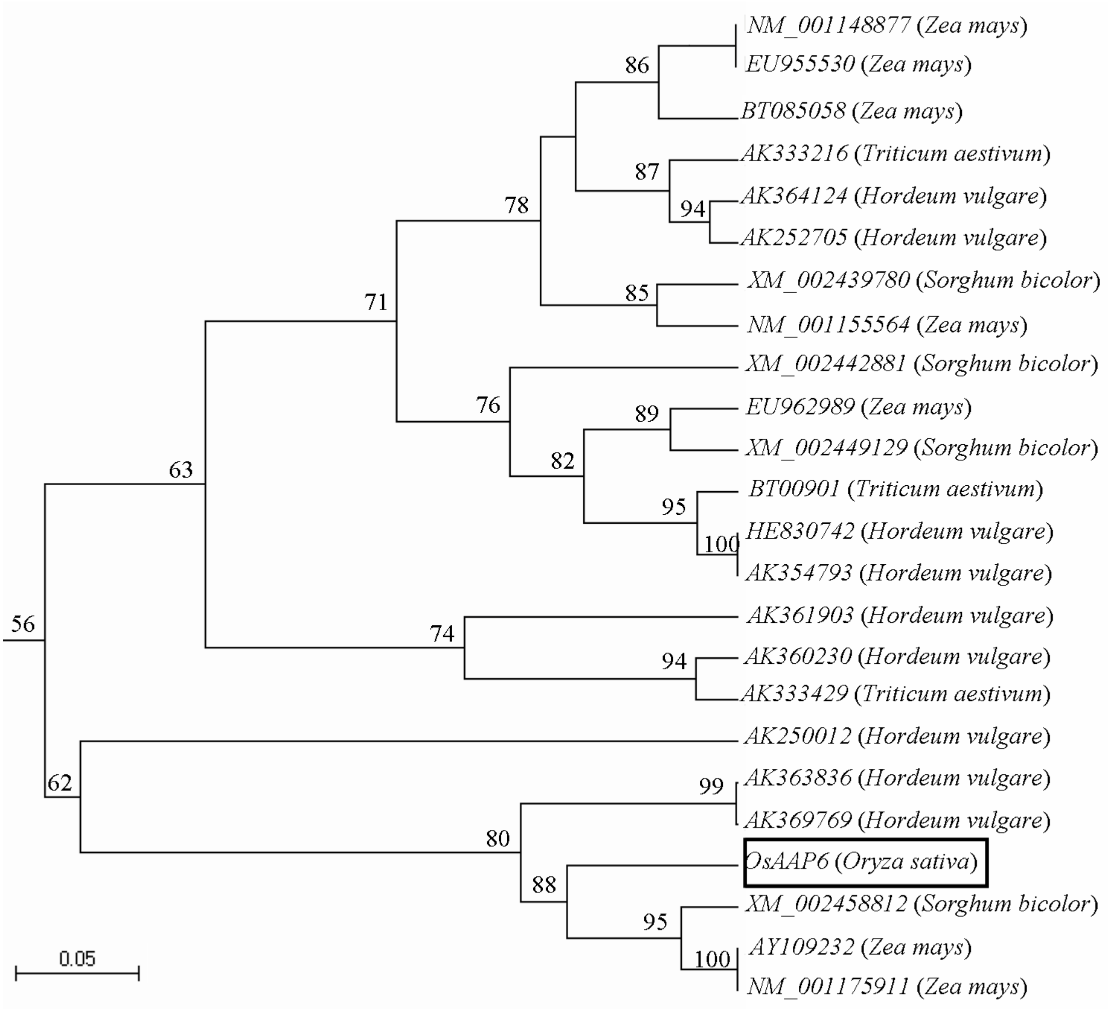


**Supplementary Figure 9 |** Phylogenetic comparison of *OsAAP6* with cDNA of other staple cereals. The phylogenetic tree of *OsAAP6* with cDNA of other staple cereals was generated by MEGA 4.0.

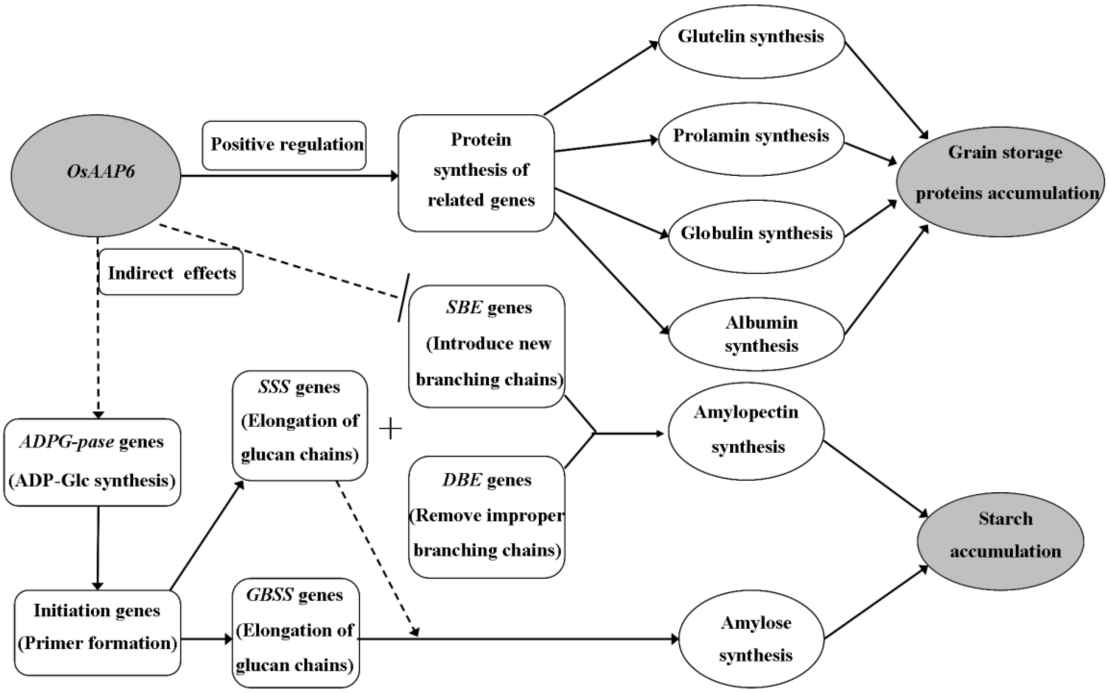


**Supplementary Figure 10 | Model for the possible role of *OsAAP6*.** ADPG-pase, ADP-glucose pyrophosphorylase; GBSS, granule-bound starch synthase; SSS, soluble starch synthase; SBE, starch branching enzyme; DBE, debranching enzyme.

**Supplementary Tables**

**Supplementary Table 1 | Protein contents of brown rice from the three genotypic classes among 320 random individuals from a $BC_3F_2$ subpopulation.**

| Genotype[a] | No. of plants | Mean±s.e.m. | Range ($mg.g^{-1}$) |
|---|---|---|---|
| ZS97 homozygotes | 83 | 120.5±1.0 (a) | 115.8-131.2 |
| Heterozygotes | 153 | 113.6±1.2 (b) | 105.5-121.1 |
| NYZ homozygotes | 84 | 108.4±0.8 (c) | 103.6-119.8 |

[a]Homozygous genotypes for the Nanyangzhan and Zhenshan 97 alleles and the heterozygous genotype were determined by SSR markers RM472 and RM104. a, b and c indicate significant differences determined by the Duncan test, $P \leq 0.01$.

**Supplementary Table 2 | Progeny tests of the six important recombinants in the interval PB11 - PB12.**

| No. | Protein content of $BC_3F_2$ progenies of each recombinant ($mg.g^{-1}$) | | | | | |
|---|---|---|---|---|---|---|
| | HL16 | HL76 | HL43 | HL84 | HL49 | HL19 |
| Mean±s.e.m. | 111.0±0.7(b) | 112.6±1.0(b) | 108.4±0.4(c) | 112.8±0.7(b) | 111.4±0.8(b) | 119.6±0.3(d) |
| Range | 101.9-115.6 | 103.7-120.3 | 103.3-112.9 | 108.1-119.2 | 106.8-116.3 | 116.5-125.0 |
| PB13 | H | H | B | H | H | A |
| *qPC1* genotype[a] | H | H | B | H | H | A |

[a]*qPC1* genotype deduced by progeny testing of more than 18 individuals from each recombinant. (b), (c) and (d) indicate significant differences determined by the Duncan test, $P \leq 0.01$.

**Supplementary Table 3 | GPC and relative expression levels of $T_2$ families from transgenic $T_1$ plants of OX(NYZ), ZpZc(NYZ) and RNAi(ZS97) obtained in a field trial grown at the Experimental Stations of Huazhong Agricultural University.**

| Family | Genotype[a] | Number of plants | Protein content[b] ($mg.g^{-1}$) | Relative expression level | Correlation coefficient |
|---|---|---|---|---|---|
| OX(NYZ) | + | 46 | 123.1±0.5 | 6.0±0.2 | 0.69 |
| | – | 22 | 109.4±0.6 | 3.5±0.4 | |
| *P*[c] | | | $6.6\times10^{-23}$ | $2.0\times10^{-8}$ | |
| ZpZc(NYZ) | + | 48 | 118.4±0.4 | 3.6±0.2 | 0.66 |
| | – | 23 | 109.2±0.4 | 2.4±0.2 | |
| *P*[c] | | | $1.3\times10^{-22}$ | $4.1\times10^{-6}$ | |
| RNAi(ZS97) | + | 41 | 103.3±0.6 | 3.2±0.1 | 0.70 |
| | – | 25 | 120.4±0.7 | 4.4±0.2 | |
| *P*[c] | | | $9.0\times10^{-24}$ | $5.7\times10^{-7}$ | |

[a]Genotypes (+) and (–) are transgene-positive and transgene-negative plants detected by *GUS* or *Hpt* PCR primers, respectively. [b]Protein contents of brown rice in $T_2$ materials; at least 300 grains of each plant were used in each measurement. [c]*P*-values produced by two-tailed *t*-test. Relative expression level of the *OsAAP6* gene in each progeny was determined by Real-Time PCR with the RNA isolated from endosperms at 10 DAF. Data are given as means ± s.e.m. $P < 0.01$ for the three correlations.

**Supplementary Table 4 | Putative *cis*-regulatory elements detected in variant regions of SNPs or the InDel between parents Zhenshen 97 and Nanyangzhan.**

| Variant region | Putative regulatory element | Putative function |
|---|---|---|
| -5 to -17 bp | Copper-response element | Target for transcriptional activators and involved in oxygen-response |
| -26 to -34 bp | Inr-element | Involved in light-responsive transcription |
| | Sulfur-responsive element | Binding sequence of auxin response factor (ARF) |
| -396 to -413 bp | HDZIP2ATATHB2 | Involved in light signals regulation; Plant polyA signal |
| -539 to -551 bp | ARR1-binding element | Binding sequence of transcriptional activator ARR1 |
| -910 to -925 bp | GATA-box | Light regulated, and involved in root hair-specific distribution |
| -1257 to -1287 bp | CAAT-box | Responsible for the tissue specific promoter activity of a pea legumin gene |
| | CCAAT-box | Acts cooperatively with heat shock elements to increase promoter activity |
| | MYBPZM | Binding site of maize myb homolog |
| | WB-box | Specifically binding sequence of WRKY proteins |
| | ARR1-binding element | Binding sequence of transcriptional activator ARR1 |
| -1381 to -1392 bp | DOFCOREZM | Binding site of Dof proteins |
| | WB-box | Specifically binding sequence of WRKY proteins |
| -1429 to -1437 bp | YACT element | *Cis*-regulatory elements for mesophyll-specific gene expression |
| | GATA-box | Light regulated, and involved in root hair-specific distribution |
| | ElRE | WRKY1 protein binding site |
| -1563 to -1572 bp | SORLIP1AT | Involved in light-induced cotyledon |

Putative plant *cis*-acting regulatory elements were predicted in the variant SNPs or InDel regions by PLACE analysis (http://www.dna.affrc.go.jp/PLACE/).

**Supplementary Table 5 | *OsAAP6* genotypes and protein contents in 197 accessions of a mini-core collection representing a broad range of rice germplasm from Asia.**

| No. | Accession | Subspecies | Sub[a] | Protein content[b] (mg.g$^{-1}$) | *OsAAP6* genotype[c] | Origin |
|---|---|---|---|---|---|---|
| 1 | Saducho | *Indica* | Sub 2 | 114.7 | Type 8 | Philippines |
| 2 | Dom Sufid | *Japonica* | Sub 2 | 115.8 | Type 5 | Philippines |
| 3 | Gerdeh | *Japonica* | Sub 2 | 113.0 | Type 6 | Iran |
| 4 | Lijiangxintuanheigu | *Japonica* | Sub 2 | 114.2 | Type 6 | China |
| 5 | Azucen A | *Japonica* | Sub 2 | 106.3 | Type 6 | Philippines |
| 6 | Shanhuangzhan 2 | *Indica* | Sub 1 | 110.3 | Type 6 | China |
| 7 | Swarna | *Indica* | Sub 1 | 105.2 | Type 2 | India |
| 8 | Moroberekan | *Japonica* | Sub 2 | 120.0 | Type 2 | Guinea |
| 9 | Cypress | *Japonica* | Sub 2 | 114.5 | Type 2 | United States |
| 10 | IR64 | *Indica* | Sub 1 | 102.8 | Type 6 | Philippines |
| 11 | Tainong 67 | *Japonica* | Sub 2 | 103.8 | Type 2 | Thailand |
| 12 | N 22 | *Aus/Boro* | Sub 2 | 117.0 | Type 6 | Philippines |
| 13 | M202 | *Japonica* | Sub 2 | 106.4 | Type 3 | Philippines |
| 14 | Dular | *Indica* | Sub 2 | 119.3 | Type 2 | India |
| 15 | Laoguangtou 83 | *Japonica* | Sub 2 | 112.4 | Type 3 | Heilongjiang, China |
| 16 | Baimaodao | *Japonica* | Sub 2 | 122.3 | Type 6 | Heilongjiang, China |
| 17 | Annongwangeng B | *Japonica* | Sub 2 | 113.4 | Type 6 | Anhui, China |
| 18 | Aijiaonante | *Indica* | Sub 1 | 118.6 | Type 2 | Zhejiang, China |
| 19 | Guangluai 4 | *Indica* | Sub 1 | 104.9 | Type 2 | Guangdong, China |
| 20 | Xiangaizao 10 | *Indica* | Sub 1 | 112.2 | Type 6 | Hunan, China |
| 21 | Geng 7623 | *Japonica* | Sub 2 | 118.5 | Type 2 | Unknown |
| 22 | Jinante B | *Indica* | Sub 1 | 115.4 | Type 2 | Hunan, China |
| 23 | Funingzipi | *Japonica* | Sub 2 | 122.2 | Type 6 | Hebei, China |
| 24 | Zhenshan 97B | *Indica* | Sub 1 | 120.9 | Type 2 | Jiangxi, China |
| 25 | Qingsiai 16B | *Indica* | Sub 1 | 112.2 | Type 2 | Guangdong, China |
| 26 | Weiguo | *Japonica* | Sub 2 | 111.6 | Type 6 | Liaoning, China |
| 27 | Zhenrui 409B | *Indica* | Sub 1 | 112.9 | Type 2 | Yunnan, China |
| 28 | Liaogeng 287 | *Japonica* | Sub 2 | 115.0 | Type 6 | Liaoning, China |
| 29 | Huhui 628 | *Japonica* | Sub 2 | 117.9 | Type 7 | Unknown |
| 30 | 88B | *Indica* | Sub 1 | 105.7 | Type 4 | Jiangsu, China |
| 31 | Xianghui 91269 | *Indica* | Sub 1 | 109.2 | Type 4 | Hunan, China |
| 32 | Longhuamaohu | *Japonica* | Sub 2 | 114.8 | Type 6 | Hebei, China |
| 33 | Lucaihao | *Indica* | Sub 1 | 110.6 | Type 2 | Fujian, China |
| 34 | Zhonglou 1 | *Japonica* | Sub 2 | 116.8 | Type 6 | Shanxi, China |
| 35 | Yelichanghua | *Japonica* | Sub 2 | 115.5 | Type 6 | Hebei, China |

| | | | | | | |
|---|---|---|---|---|---|---|
| 36 | Shufeng101 | *Indica* | Sub 1 | 113.5 | Type 2 | Sichuan, China |
| 37 | Chengduai 3 | *Indica* | Sub 1 | 108.2 | Type 4 | Sichuan, China |
| 38 | Sankecun | *Indica* | Sub 1 | 107.2 | Type 4 | Hunan, China |
| 39 | Gongju 73 | *Japonica* | Sub 1 | 107.8 | Type 6 | Yunnan, China |
| 40 | Jiabala | *Indica* | Sub 1 | 105.3 | Type 4 | Xizang, China |
| 41 | Taishanruo | *Indica* | Sub 1 | 115.3 | Type 2 | Guangdong, China |
| 42 | Guicao 2 | *Indica* | Sub 1 | 108.4 | Type 2 | Guangdong, China |
| 43 | Huke 3 | *Indica* | Sub 1 | 104.4 | Type 6 | Shanghai, China |
| 44 | Teqingxuanhui | *Indica* | Sub 1 | 102.3 | Type 2 | Unknown |
| 45 | Huangsiguizhan | *Indica* | Sub 1 | 108.1 | Type 6 | Guangdong, China |
| 46 | Xiangwanxian 3 | *Indica* | Sub 1 | 107.1 | Type 4 | Hunan, China |
| 47 | Taizhong 65 | *Japonica* | Sub 2 | 110.4 | Type 2 | Thailand |
| 48 | Zaoshunonghu 6 | *Japonica* | Sub 2 | 106.3 | Type 6 | Hunan, China |
| 49 | Jinyou 1 | *Indica* | Sub 1 | 103.5 | Type 6 | Fujian, China |
| 50 | Chengnongshuijing | *Indica* | Sub 1 | 103.7 | Type 6 | Sichuan, China |
| 51 | PeiC122 | *Japonica* | Sub 2 | 108.3 | Type 2 | Unknown |
| 52 | Guihuahuang | *Japonica* | Sub 2 | 106.6 | Type 6 | Jiangsu, China |
| 53 | Momi | *Indica* | Sub 1 | 101.8 | Type 6 | Guangxi, China |
| 54 | Xiushui 115 | *Japonica* | Sub 2 | 98.5 | Type 6 | Zhejiang, China |
| 55 | Sanbaili | *Indica* | Sub 1 | 109.1 | Type 2 | Hunan, China |
| 56 | Pudao 1 | *Japonica* | Sub 2 | 109.7 | Type 6 | Shanxi, China |
| 57 | Dandongludao | *Japonica* | Sub 2 | 111.3 | Type 6 | Liaoning, China |
| 58 | Liusha 1 | *Indica* | Sub 1 | 111.9 | Type 2 | Guangxi, China |
| 59 | Bawangbian 1 | *Japonica* | Sub 1 | 108.7 | Type 4 | Hubei, China |
| 60 | Yangdao 2 | *Indica* | Sub 1 | 102.7 | Type 6 | Jiangsu, China |
| 61 | Zhengdao 5 | *Japonica* | Sub 2 | 107.0 | Type 2 | Henan, China |
| 62 | Geng87-304 | *Japonica* | Sub 2 | 93.2 | Type 6 | Hunan, China |
| 63 | Sugeng 2 | *Japonica* | Sub 2 | 100.3 | Type 6 | Jiangsu, China |
| 64 | Hongwan 1 | *Indica* | Sub 1 | 101.5 | Type 6 | Fujian, China |
| 65 | Liushizao | *Indica* | Sub 1 | 108.6 | Type 2 | Sichuan, China |
| 66 | Muxiqiu | *Japonica* | Sub 2 | 109.2 | Type 6 | Jiangsu, China |
| 67 | Taizhongxianxuan 2 | *Indica* | Sub 1 | 109.5 | Type 2 | Thailand |
| 68 | Dangyu 5 | *Japonica* | Sub 1 | 105.4 | Type 4 | Anhui, China |
| 69 | Youmangzaogeng | *Japonica* | Sub 2 | 115.6 | Type 6 | Jiangsu, China |
| 70 | Laohuzhong | *Japonica* | Sub 2 | 95.8 | Type 6 | Shanghai, China |
| 71 | Nantehao | *Indica* | Sub 1 | 109.1 | Type 2 | Jiangxi, China |
| 72 | Ersiniu | *Indica* | Sub 2 | 114.1 | Type 2 | Guangdong, China |
| 73 | Cunsanli | *Japonica* | Sub 2 | 115.8 | Type 6 | Jiangsu, China |
| 74 | Huangkezaogan | *Japonica* | Sub 2 | 116.0 | Type 6 | Jiangsu, China |
| 75 | Nantiangangjiu | *Japonica* | Sub 2 | 112.1 | Type 2 | Sichuan, China |
| 76 | Benbanggu | *Japonica* | Sub 2 | 114.0 | Type 2 | Yunnan, China |
| 77 | Qitoubaigu | *Japonica* | Sub 1 | 121.2 | Type 2 | Yunnan, China |
| 78 | Muguaruo | *Japonica* | Sub 2 | 114.0 | Type 6 | Hunan, China |
| 79 | Hanmadao | *Indica* | Sub 1 | 110.1 | Type 2 | Henan, China |

| | | | | | | |
|---|---|---|---|---|---|---|
| 80 | Heidu 4 | *Indica* | Sub 1 | 114.9 | Type 2 | Guangdong, China |
| 81 | Gaoyangdiandao | *Japonica* | Sub 2 | 117.3 | Type 6 | Hebei, China |
| 82 | Chikenuo | *Indica* | Sub 2 | 115.1 | Type 6 | Fujian, China |
| 83 | Haobuke | *Japonica* | Sub 2 | 124.7 | Type 6 | Yunnan, China |
| 84 | Shanjiugu | *Japonica* | Sub 2 | 119.1 | Type 6 | Sichuan, China |
| 85 | Fanhaopi | *Indica* | Sub 1 | 110.6 | Type 2 | Yunnan, China |
| 86 | Lixingeng | *Japonica* | Sub 2 | 105.1 | Type 6 | Sichuan, China |
| 87 | Baigegu | *Japonica* | Sub 2 | 117.9 | Type 2 | Jiangsu, China |
| 88 | Tieganniao | *Japonica* | Sub 2 | 99.7 | Type 6 | Zhejiang, China |
| 89 | Shancunli | *Japonica* | Sub 2 | 106.4 | Type 2 | Shanxi, China |
| 90 | Meihuaruo | *Indica* | Sub 1 | 108.0 | Type 2 | Sichuan, China |
| 91 | Lamujia | *Japonica* | Sub 2 | 108.2 | Type 4 | Yunnan, China |
| 92 | Maguruo | *Japonica* | Sub 2 | 110.5 | Type 6 | Guizhou, China |
| 93 | Menjiagao 1 | *Japonica* | Sub 1 | 107.1 | Type 4 | Hainan, China |
| 94 | Xiaohonggu | *Japonica* | Sub 1 | 104.2 | Type 6 | Yunnan, China |
| 95 | Jinxibai | *Indica* | Sub 1 | 105.6 | Type 4 | Jiangxi, China |
| 96 | Jinbaoyin | *Japonica* | Sub 1 | 107.6 | Type 2 | Fujian, China |
| 97 | Hongairuo | *Indica* | Sub 1 | 107.8 | Type 4 | Guangxi, China |
| 98 | Aizizhan | *Indica* | Sub 1 | 114.0 | Type 2 | Guangxi, China |
| 99 | Babaili | *Japonica* | Sub 2 | 107.5 | Type 6 | Yunnan, China |
| 100 | Qiyuexian | *Indica* | Sub 1 | 113.1 | Type 2 | Guangxi, China |
| 101 | Mamagu | *Indica* | Sub 1 | 106.1 | Type 4 | Sichuan, China |
| 102 | Qingke | *Indica* | Sub 1 | 106.9 | Type 4 | Yunnan, China |
| 103 | Hongkezheruo | *Japonica* | Sub 2 | 110.1 | Type 6 | Guizhou, China |
| 104 | Mowangunei | *Japonica* | Sub 2 | 107.3 | Type 2 | Yunnan, China |
| 105 | Honggu | *Indica* | Sub 2 | 112.1 | Type 2 | Sichuan, China |
| 106 | Zimi | *Japonica* | Sub 1 | 116.5 | Type 2 | Yunnan, China |
| 107 | Xianggu | *Japonica* | Sub 1 | 114.4 | Type 2 | Yunnan, China |
| 108 | Ximaxian | *Indica* | Sub 2 | 114.9 | Type 6 | Yunnan, China |
| 109 | Jinnante 43 | *Indica* | Sub 1 | 116.0 | Type 2 | Hunan, China |
| 110 | Xiangzaoxian 7 | *Indica* | Sub 1 | 113.7 | Type 2 | Hunan, China |
| 111 | 80B | *Indica* | Sub 1 | 114.3 | Type 2 | Hunan, China |
| 112 | Baoxie123B | *Indica* | Sub 1 | 115.0 | Type 2 | Hunan, China |
| 113 | LimingB | *Japonica* | Sub 2 | 108.9 | Type 6 | Liaoning, China |
| 114 | Jiangnongzao 1 | *Indica* | Sub 1 | 118.4 | Type 2 | Jiangxi, China |
| 115 | Gu154 | *Indica* | Sub 1 | 106.5 | Type 4 | Unknown |
| 116 | Ninghui 21 | *Japonica* | Sub 2 | 104.6 | Type 2 | Unknown |
| 117 | Shuiyuan 300li | *Japonica* | Sub 2 | 111.0 | Type 6 | Tianjin, China |
| 118 | XiangaiB | *Indica* | Sub 1 | 105.4 | Type 4 | Jiangxi, China |
| 119 | Aituogu 151 | *Indica* | Sub 1 | 109.3 | Type 2 | Sichuan, China |
| 120 | Taidongluai | *Japonica* | Sub 2 | 112.7 | Type 1 | Thailand |
| 121 | Menjiading 2 | *Indica* | Sub 1 | 112.7 | Type 2 | Hainan, China |
| 122 | Jiefangxian | *Indica* | Sub 1 | 109.9 | Type 4 | Jiangxi, China |
| 123 | Hongqi 5 | *Japonica* | Sub 2 | 109.2 | Type 6 | Hunan, China |

| | | | | | | |
|---|---|---|---|---|---|---|
| 124 | Baikwhualuo | *Indica* | Sub 1 | 116.8 | Type 2 | Guangdong, China |
| 125 | Liuyenian | *Indica* | Sub 1 | 110.1 | Type 2 | Hubei, China |
| 126 | Aihechi | *Indica* | Sub 1 | 99.6 | Type 8 | Jiangxi, China |
| 127 | Xiangruo | *Japonica* | Sub 2 | 114.2 | Type 6 | Guizhou, China |
| 128 | Xuanenchangtan | *Indica* | Sub 1 | 112.3 | Type 2 | Hubei, China |
| 129 | Jinzhiruo | *Indica* | Sub 1 | 107.2 | Type 4 | Yunnan, China |
| 130 | Laohongdao | *Japonica* | Sub 2 | 113.3 | Type 6 | Shanxi, China |
| 131 | Wuzidui | *Japonica* | Sub 2 | 117.3 | Type 6 | Yunnan, China |
| 132 | Xibainian | *Japonica* | Sub 2 | 116.8 | Type 2 | Sichuan, China |
| 133 | Zhegu | *Japonica* | Sub 1 | 104.4 | Type 6 | Guizhou, China |
| 134 | Cunguruo | *Indica* | Sub 2 | 118.3 | Type 6 | Guizhou, China |
| 135 | Lengshuigu 2 | *Japonica* | Sub 2 | 106.3 | Type 6 | Yunnan, China |
| 136 | Banjiemang | *Japonica* | Sub 2 | 110.8 | Type 2 | Yunnan, China |
| 137 | Wujuhonggu | *Indica* | Sub 1 | 116.9 | Type 2 | Yunnan, China |
| 138 | Nangaogu | *Indica* | Sub 1 | 113.5 | Type 2 | Yunnan, China |
| 139 | Mowanggunei | *Japonica* | Sub 2 | 113.4 | Type 2 | Yunnan, China |
| 140 | Qitougu | *Indica* | Sub 1 | 113.8 | Type 2 | Yunnan, China |
| 141 | Huangpiruo | *Japonica* | Sub 2 | 111.8 | Type 6 | Yunnan, China |
| 142 | Zhenshan 97 | *Indica* | Sub 1 | 121.4 | Type 2 | Zhejiang, China |
| 143 | Nipponare | *Japonica* | Sub 2 | 108.5 | Type 6 | Japan, China |
| 144 | Minghui 63 | *Indica* | Sub 1 | 108.9 | Type 4 | Fujian, China |
| 145 | 9311 | *Indica* | Sub 1 | 112.1 | Type 2 | Jiangsu, China |
| 146 | Zhonghua 11 | *Japonica* | Sub 2 | 117.1 | Type 6 | Beijing, China |
| 147 | Bllila | *Japonica* | Sub 2 | 111.4 | Type 6 | Italy |
| 148 | 2428 | *Japonica* | Sub 2 | 105.0 | Type 2 | Jiangsu, China |
| 149 | IRAT109 | *Japonica* | Sub 2 | 109.8 | Type 6 | Cote d'ivoire |
| 150 | Heigeng 2 | *Japonica* | Sub 2 | 124.9 | Type 6 | Heilongjiang, China |
| 151 | Erjiunan 1 | *Indica* | Sub 1 | 119.6 | Type 2 | Zhejiang, China |
| 152 | Zhaoyang 1B | *Indica* | Sub 1 | 118.6 | Type 2 | Hunan, China |
| 153 | L301B | *Indica* | Sub 1 | 115.3 | Type 2 | Hunan, China |
| 154 | Guangluai 15 | *Indica* | Sub 1 | 111.4 | Type 2 | Guangxi, China |
| 155 | Zhuzhen B | *Indica* | Sub 1 | 115.4 | Type 2 | Hunan, China |
| 156 | Baoxie 7B | *Indica* | Sub 1 | 117.4 | Type 2 | Hunan, China |
| 157 | Gzhenshan 97B | *Japonica* | Sub 2 | 117.1 | Type 2 | Sichuan, China |
| 158 | nanxiongzaoyou | *Indica* | Sub 1 | 108.4 | Type 2 | Guangdong, China |
| 159 | Zaoshuxianghei | *Indica* | Sub 1 | 109.9 | Type 4 | Guangxi, China |
| 160 | IR661-1 | *Indica* | Sub 1 | 113.3 | Type 2 | Unknown |
| 161 | Gui 630 | *Indica* | Sub 1 | 110.5 | Type 2 | Unknown |
| 162 | Jan-76 | *Japonica* | Sub 2 | 109.7 | Type 2 | Unknown |
| 163 | Xuguruo | *Japonica* | Sub 1 | 110.2 | Type 2 | Hunan, China |
| 164 | Taizhongzailai 1 | *Indica* | Sub 1 | 110.8 | Type 2 | Thailand |
| 165 | Xiangwanxian 1 | *Indica* | Sub 1 | 115.4 | Type 2 | Hunan, China |
| 166 | Xingguo | *Japonica* | Sub 2 | 112.7 | Type 6 | Jilin, China |
| 167 | Zhonghua 8 | *Japonica* | Sub 2 | 114.1 | Type 6 | Beijing, China |

| | | | | | | |
|---|---|---|---|---|---|---|
| 168 | Aimakang | *Indica* | Sub 1 | 108.8 | Type 4 | Sichuan, China |
| 169 | JWR221 | *Japonica* | Sub 1 | 111.3 | Type 2 | Unknown |
| 170 | Zhenxian 232 | *Indica* | Sub 1 | 115.2 | Type 2 | Jiangsu, China |
| 171 | Chenwan 3 | *Japonica* | Sub 1 | 106.6 | Type 5 | Hunan, China |
| 172 | Maguzi | *Japonica* | Sub 2 | 116.5 | Type 6 | Shanxi, China |
| 173 | Aimi | *Indica* | Sub 1 | 110.2 | Type 2 | Jiangxi, China |
| 174 | Haobayong 1 | *Japonica* | Sub 2 | 105.4 | Type 6 | Yunnan, China |
| 175 | Wanlixian | *Indica* | Sub 1 | 115.1 | Type 2 | Hunan, China |
| 176 | Feidongtangdao | *Japonica* | Sub 2 | 116.1 | Type 6 | Anhui, China |
| 177 | Hengxianliangchun | *Indica* | Sub 1 | 112.7 | Type 2 | Guangxi, China |
| 178 | Leihuozhan | *Indica* | Sub 1 | 115.8 | Type 2 | Anhui, China |
| 179 | Baikezaohe | *Indica* | Sub 1 | 105.1 | Type 4 | Hunan, China |
| 180 | Haomake | *Japonica* | Sub 2 | 111.3 | Type 6 | Yunnan, China |
| 181 | Sanqishi | *Japonica* | Sub 2 | 111.3 | Type 4 | Yunnan, China |
| 182 | Niankeruo | *Indica* | Sub 2 | 112.4 | Type 4 | Guizhou, China |
| 183 | Yangkeruo | *Indica* | Sub 2 | 117.9 | Type 4 | Guizhou, China |
| 184 | Putaohuang | *Japonica* | Sub 2 | 120.7 | Type 4 | Tianjin, China |
| 185 | Xiangdao | *Indica* | Sub 1 | 107.9 | Type 4 | Henan, China |
| 186 | Younian | *Japonica* | Sub 1 | 112.1 | Type 2 | Guizhou, China |
| 187 | Wukezhan | *Indica* | Sub 1 | 124.1 | Type 2 | Fujian, China |
| 188 | Zhongnong 4 | *Indica* | Sub 1 | 120.9 | Type 2 | Sichuan, China |
| 189 | Mibeiwanxian | *Indica* | Sub 1 | 108.0 | Type 4 | Fujian, China |
| 190 | Hongmmishandan | *Japonica* | Sub 2 | 116.4 | Type 6 | Jiangxi, China |
| 191 | Maweinian | *Indica* | Sub 1 | 110.6 | Type 2 | Guizhou, China |
| 192 | Ziruo | *Indica* | Sub 1 | 119.0 | Type 2 | Yunnan, China |
| 193 | Beiziruo | *Japonica* | Sub 1 | 107.0 | Type 4 | Yunnan, China |
| 194 | Pwusheng | *Indica* | Sub 1 | 106.8 | Type 1 | Yunnan, China |
| 195 | Yizhixiang | *Indica* | Sub 1 | 109.0 | Type 4 | Fujian, China |
| 196 | Xiaobaimi | *Indica* | Sub 1 | 119.3 | Type 2 | Guizhou, China |
| 197 | Zaoxian 240 | *Indica* | Sub 1 | 126.1 | Type 2 | Anhui, China |

[a]Sub, subpopulation; and Sub1 and Sub2 were classified by population structure analysis as described previously, the population structure of the mini-core collection of 197 accessions was investigated by the model-based method implemented in STRUCTURE followed the procedure described[32,33]. [b]Protein content was measured using phenotypic data from years of 2010 and 2011 in Wuhan. At least 300 grains for each accession at each year were measured. The correlation coefficient for protein content of brown rice between the two years is 0.75 ($P < 0.01$). [c]*OsAAP6* genotype is determined and grouped by sequencing the 5'-UTR and promoter region and coding region of *OsAAP6* in the accessions, as shown in **Fig. 3b**.

**Supplementary Table 6 | Yield and yield component traits of near-isogenic lines (NILs) and transgenic plants.**

| Trait | No. of plants | 1,000-grain weight (g) | Grain weight per plant (g) | Number of panicles per plant | Number of grains per panicle | Plant height (cm) |
|---|---|---|---|---|---|---|
| NIL(ZS97) | 36 | 24.35±0.21 | 19.78±0.36 | 9.9±0.22 | 79.8±3.4 | 82.42±0.55 |
| NIL(NYZ) | 38 | 24.23±0.16 | 18.58±0.40 | 9.5±0.18 | 77.8±2.5 | 82.56±0.56 |
| *P*[a] | | 0.48 | 0.06 | 0.07 | 0.46 | 0.77 |
| OX(NYZ)– | 24 | 36.58±0.26 | 11.27±0.37 | 5.6±0.21 | 86.7±2.7 | 150.29±0.71 |
| OX(NYZ)+ | 20 | 36.19±0.29 | 11.40±0.41 | 5.5±0.23 | 84.1±3.3 | 149.58±0.92 |
| *P*[a] | | 0.65 | 0.70 | 0.61 | 0.35 | 0.35 |
| OX(ZH11)– | 24 | 22.92±0.27 | 17.56±0.44 | 7.5±0.18 | 123.9±3.4 | 96.82±1.11 |
| OX(ZH11)+ | 24 | 22.78±0.21 | 16.83±0.38 | 7.3±0.25 | 122.6±4.1 | 95.95±0.91 |
| *P*[a] | | 0.52 | 0.10 | 0.32 | 0.69 | 0.35 |
| ZpZc(NYZ)– | 24 | 36.18±0.24 | 11.31±0.35 | 5.4±0.19 | 85.7±2.1 | 150.17±0.78 |
| ZpZc(NYZ)+ | 22 | 36.22±0.19 | 11.43±0.46 | 5.5±0.26 | 86.6±2.9 | 149.82±0.96 |
| *P*[a] | | 0.83 | 0.74 | 0.62 | 0.69 | 0.65 |
| RNAi(ZS97)– | 24 | 24.31±0.19 | 19.26±0.32 | 9.8±0.21 | 79.1±3.0 | 82.39±0.75 |
| RNAi(ZS97)+ | 24 | 24.17±0.27 | 18.69±0.39 | 9.6±0.19 | 78.6±2.7 | 82.28±0.78 |
| *P*[a] | | 0.50 | 0.12 | 0.29 | 0.84 | 0.87 |
| RNAi(ZH11)– | 24 | 23.02±0.25 | 17.59±0.39 | 7.4±0.26 | 122.7±3.5 | 96.73±1.21 |
| RNAi(ZH11)+ | 24 | 22.76±0.28 | 17.83±0.50 | 7.6±0.23 | 121.9±3.7 | 95.66±1.18 |
| *P*[a] | | 0.30 | 0.55 | 0.37 | 0.80 | 0.33 |

[a]*P*-values produced by two-tailed *t*-test. All data are means ± s.e.m. (+) and (–) indicate transgene-positive and negative plants, respectively.

**Supplementary Table 7 | Primer sets used for qRT-PCR of the 81 genes involved in metabolism of storage starch and grain storage proteins.**

| FL-cDNA | Gene | Forward primer (5'-3')[a] | Reverse primer (5'-3') | Annotated name |
|---|---|---|---|---|
| AK108254 | *10KD Prolamin* | TGCAGTATTTCCCACCAACA | ACATGAACATGGCTGTGGAG | 10 Kda prolamin |
| AK121775 | *11S Globulin* | CACCAAACCCGATCTTCAGT | CGGAACAGCTTCTCCATCTC | 11S globulin |
| AK242306 | *13KD Prolamin* | CACAGCGCAGTTTGATGTTT | GCTTGCCGCAATGCTATACT | 13 Kda prolamin |
| AK242322 | *17KD Prolamin* | TTTGATGCTTGCACCTATGG | GCAGCTGCTCAGTTTTAGCC | 17 Kda prolamin |
| AK287940 | *19KD Globulin* | GCCAGTAATTGCAGGGGATA | AGGTCACCACCAACGTAAGC | 19 Kda globulin precursor (α-globulin) |
| AK100910 | *AGPL1* | CATCAAGGACGGGAAGGTCA | ACTTCACTCGGGGCAGCTTA | ADP-glucose pyrophosphorylase large subunit 1 |
| AK069296 | *AGPL3* | GACCATTTGCGGCAGGAATA | TGGAACAACCAATACCCCAGA | ADP-glucose pyrophosphorylase large subunit 3 |
| AK121036 | *AGPL4* | TCCCTTCTGGTTTGTTGCATTT | CCAAAACGTTCTTGCCATGC | ADP-glucose pyrophosphorylase large subunit 4 |
| AK073146 | *AGPS1* | AGAATGCTCGTATTGGAGAAAATG | GGCAGCATGGAATAAACCAC | ADP-glucose pyrophosphorylase small subunit 1 |
| AK071826 | *AGPS2a* | AGTAGTGGGACTCCGGTCCT | ATGCCACCTTTTTCACCAAG | ADP-glucose pyrophosphorylase small subunit 2a |
| AK103906 | *AGPS3b* | AACAATCGAAGCGCGAGAAA | GCCTGTAGTTGGCACCCAGA | ADP-glucose pyrophosphorylase small subunit 2b |
| AK102488 | *AlaAT* | CCATTTCTCGAGCAACAACA | ATTTGCAGGGTATCCGTCAC | Alanine aminotransferase |
| AK068061 | *G6PIa* | TCAGCATGCCTACATTCAGC | TGCAACATCCCGAACAAATA | Glucose-6-phosphate isomerase A |
| AK068236 | *G6PIb* | GTTGCACGAAGCATCAAAGA | ATGTTTCGCAACAGCATCAG | Glucose-6-phosphate isomerase, cytosolic B |
| AK107343 | *GluB1* | GCCAAAGTCAGAGCCAAAAG | GAACCAATGTGCAACACCAG | Glutelin B1 |
| AK243000 | *Glutelinλ* | TGGCGACCATAGCTTTCTCT | GGGTTGTGCCATGGATTTAC | Glutelin λ-RG21 |
| AK242245 | *GluA1* | CATTTGAGCCAATTCGGAGT | GGCCTGATTGTTGGAACTGT | Glutelin A1 |
| AK107314 | *GluA2* | GCAAGAGCAGGAACAAGGAC | CCTCATGGTGCAAAAGGTCT | Glutelin A2 |
| AK107271 | *GluA3* | TGAAAACCAACCCTGACTCC | ACTCATCTCCCCTGTTGTGC | Glutelin A3 |
| AK242872 | *GluB4* | GCGACCAGAAGGCTACAAAG | TTGCTTGTTGATCGTTGCTC | Glutelin B4 |
| AK070431 | *GBSSI* | TCCGAGAGGTTCAGGTCATC | ATGAGCTCCTCGGCGTAGTA | Granule-bound starch synthase I |
| AK067654 | *GBSSII* | AAACGGGCTCTGAAGCAGTA | CTCCTCCCACTTCTTTGCAG | Granule-bound starch synthase II |
| AK105347 | *Globulin1* | ATCGAGAACGGCGAGAAGT | GGACGGAGATGGTATGGAGA | Homolog of maize globulin 1 |
| AK121667 | *Globulin2* | CGACGAGGTGTTCTACGTCA | GTGTTGGCGGAGTAGACGAT | Homolog of maize globulin 2 |
| AB093426 | *ISA1* | TGCTCAGCTACTCCTCCATCATC | AGGACCGCACAACTTCAACATA | Isoamylase I |
| AC132483 | *ISA2* | TAGAGGTCCTCTTGGAGG | AATCAGCTTCTGAGTCACCG | Isoamylase II |
| AP005574 | *ISA3* | ACAGCTTGAGACACTGGGTTGAG | GCATCAAGAGGACAACCATCTG | Isoamylase III |
| AK068268 | *PDI* | ACCAGGGCAAGAACATTCAG | TTGCGTCTTCTGGTGACTTG | Protein disulfide-isomerase precursor |
| AB012915 | *Pullulanase* | ACCTTTCTTCCATGCTGG | CAAAGGTCTGAAAGATGGG | Pullulanase |
| AK242298 | *RA16* | AGGTAGTGATCTCGGCGTTG | CCGATTCCTGGCTGACATAG | Allergen protein RA16 |
| AK242340 | *RA17* | TTCTCGGTATTGCTCCTCGT | CTTATTCCTGGCCGACATTG | Allergen protein RA17 |
| AK242333 | *RA5B* | TGGCTTCCAACAAGGTAGTG | ACCTGGTCTTGGTGGTGGTA | Allergen protein RA5B |
| AK107328 | *RAG2* | AGGTAGTGTTCTCGGCGTTG | GTACATCGGGTAGCCCATTC | Seed allergen RAG2 (RA14b) |
| AK119436 | *SBE1* | GGCATTGCACTCCAAAAGAT | GCTCCAGTTGTTGCCTTCTC | Starch branching enzyme I |
| AB023498 | *SBEIIa* | GCCAATGCCAGGAAGATGA | GCGCAACATAGGATGGGTTT | Starch branching enzyme IIa |
| D16201 | *SBEIIb* | ATGCTAGAGTTTGACCGC | AGTGTGATGGATCCTGCC | Starch branching enzyme IIb |
| AK109458 | *SSI* | TCATGGATGTGAAGGAGCAA | TGGCAGTGAACCACAAACAT | Starch synthase I |
| AK101978 | *SSIIa* | GATCGACCAGGATGACGATT | GGGTAAAGCACCTGCAACAT | Starch synthase IIa |
| AK066446 | *SSIIb* | AGGACGTGCAGGTGGTGAT | ACCTTGTCGTTGTGCTCCGA | Starch synthase IIb |

| | | | | |
|---|---|---|---|---|
| **AK072339** | ***SSIIc*** | **CGTGGCCCATTAGATGACTT** | **CAGTAAGCAAACGGTCAGCA** | **Starch synthase IIc** |
| **AK061604** | ***SSIIIa*** | **GCCTGCCCTGGACTACATTG** | **GCAAACATATGTACACGGTTCTGG** | **Starch synthase IIIa** |
| **AK122098** | ***SSIIIb*** | **ATTCCGCTCGCAAGAACTGA** | **CAACCGCAGGATAACGGAAA** | **Starch synthase IIIb** |
| **AK103906** | ***SSIVa*** | **GGGAGCGGCTCAAACATAAA** | **CCGTGCACTGACTGCAAAAT** | **Starch synthase IVa** |
| **AK067577** | ***SSIVb*** | **GAGCTGCTCCTGCTCAAGAT** | **ACACAATTGCACCCTTGACA** | **Starch synthase IVb** |
| **AK100546** | ***Susy1*** | **AATGGTATCCTCCGCAAGTG** | **GGCTTGCATTTCCCTCATAA** | **Sucrose synthase 1** |
| **AK100334** | ***Susy2*** | **GCTGAAGGACAGGAACAAGC** | **CACCACAGACAACCACAAGG** | **Sucrose synthase 2** |
| **AK289078** | ***Susy3*** | **CATGTACCCCCTGCTCAACT** | **GTCAGCTGTAATGCCTGCAA** | **Sucrose synthase 3** |
| **AK099330** | ***Amy3A*** | **CCCAGGAGTACCATGCATCT** | **CTTGGTGATGACCCTCTCGT** | **α-amylase 3A** |
| **AK241191** | ***Amy3B*** | **AGCGGTCTCAGAGTTCCTGCA** | **TCAAATCTTATTCCAGGCACCA** | **α-amylase 3B** |
| **AK101358** | ***Amy3C*** | **CTGGCTCCACACAGAACTCA** | **CGTAGACATCTCCGTCAGCA** | **α-amylase 3C** |
| **AK119761** | ***Amy3D*** | **GTCGACGAGAAGGTCATGGT** | **CCTTCTCCCAGACGCTGTAG** | **α-amylase 3D** |
| **AK064300** | ***Amy3E*** | **ACAAGGTCATGCAGGGCTAC** | **GTTCCTTGACCGGATTTCAG** | **α-amylase 3E** |
| **AK071497** | ***AGPL2*** | **CTGAGGAAGAGGTGCTTTGG** | **TCTTTCGGGAGGATTGTGTC** | **ADP-glucose pyrophosphorylase large subunit 2** |
| **AK103898** | ***FLO4e*** | **CATGCACTGTTCGAGGAGAA** | **GGGAAATGGCTCTCCCTTAG** | **Pyruvate, phosphate dikinase 1 (OsPPDKB)** |
| **Os04g0645100** | ***FLO2*** | **CACACCCTCCAGCAATATCA** | **CCTTCTGCGACTGCTTTTCT** | **FLO2** |
| **AK100306** | ***Susy3*** | **CACCAACCTCGACAGGCGTGA** | **TGGCCAATGGAACGGTGGTAGC** | **Sucrose synthase 3** |
| **AK242706** | ***TPS*** | **CGGATTTCGATATCAAGTC** | **CAAGCAAGGTAGCTTCTTC** | **Trehalose-6-phosphate synthase** |
| **AB115915** | ***SSS2-3*** | **GCACACTGCACTCCTGCCTGTT** | **GGAATTCATCTACTGGGCCACG** | **Soluble starch synthase 2-3, chloroplast precursor** |
| **AK241901** | ***Prolamin*** | **TCTCCAACCAACAATAGCAATG** | **TGCGTAGCTATCTGTGCCCGTC** | **Prolamin** |
| **AK060618** | ***ISA*** | **CAACCGTGCAATCCTCACAC** | **CAAATGGTAAGCGGTTGGACT** | **Isoamylase** |
| **AK242259** | ***RCD1*** | **GGTTTCGCTGCACTGCAGA** | **CCACTTGTCATCTTAACCAGCA** | **Cell differentiation protein rcd1** |
| **AK071514** | ***PDI1-4*** | **GAAGCACGCTAGCATCCCTT** | **GAAGCTGTGCTCTGATCCTTC** | **OsPDIL1-4 - Oryza sativa protein disulfide isomerase** |
| **AY506572** | ***MAPK4*** | **CAGATGATCATCCATCCACTC** | **TGTCAGGGTGTCGCTGACAAGC** | **Mitogen-activated protein kinase homolog 4** |
| **AK065247** | ***APS*** | **CTCTACTTCTTGAGCGTCGT** | **TCAATCTCTTCCAGAGAAC** | **Arabinose-proton symporter** |
| **AK107226** | ***Fucosidase2*** | **CATCCTGCACCACTAGAACCA** | **CTTGGTTTGGTTCATGCATCC** | **Alpha-L-fucosidase 2 precursor** |
| **AK110517** | ***Csa*** | **CACGGATCCGATTCCTCCT** | **TCCAGATCACCCTTTCTTA** | **Cellulose synthase-like family A; mannan synthase** |
| **AK108277** | ***DRP*** | **GGTGGCAGAGCTCACCGAC** | **AGCTGCTCTGGCACGATGAG** | **Desiccation-related protein PCC13-62 precursor** |
| **AK101770** | ***SMP*** | **CTCACCATCCCTGCATTGGT** | **TTCCATGCTTGTCCACATGC** | **Seed maturation protein** |
| **AK243420** | ***ATPase 2*** | **AGGCTGGTGAACGCGTT** | **TCAGACTACCACTCCATTGT** | **ATPase 2, putative, expressed** |
| **AK100992** | ***Fucosidase2*** | **AAGCTGCCCTTCAGCTACTTC** | **TGGTTCATGGATGATCCTC** | **Alpha-L-fucosidase 2 precursor** |
| **AK242312** | ***Glutelin4*** | **GTCTAGACGAGATTATGT** | **CCTTGTGATCCTTCCAC** | **Glutelin type-B 4 precursor** |
| **AK063766** | ***GPI*** | **TGGTGCTGAGGCACATGAA** | **CACTGCGGACAAATCTCTT** | **Alpha-1,4 glucan phosphorylase, chloroplast precursor** |
| **AK068629** | ***Importin7*** | **CATCTGATCCTATCAGGTTCCA** | **GTTGAGCTATGCCACTAGCAAG** | **Importin-7** |
| **AK103944** | ***PDI2-1*** | **GATCATGGAGAAGGGCTCTGA** | **CTTCTCCAACATGCGTTGAAGCC** | **Oryza sativa protein disulfide isomerase** |
| **AK069501** | ***GST*** | **CATATGCACATGATGATGTTAG** | **AGCTGAACACAAAGCTCCTGAG** | **Beta-1,3-galactosyltransferase sqv-2** |
| **AK069188** | ***V-ATPase*** | **GTATGACAATCTTGTTGTGA** | **ACCCAGTGTAGACGAAGTG** | **Vacuolar ATP synthase 91 kDa subunit** |
| **AK104070** | ***ABP*** | **GTCACCACGCGAGGCAATACTG** | **AGAGCAGATGCATGCGCTCAAC** | **ATP binding protein** |
| **AK101816** | ***ATPase*** | **GTCCATCCTATTCGACCTGAG** | **CTTTGCGATGATGGTTCCAAG** | **ATPase** |
| **AK058216** | ***ATPase*** | **TGTTCGATGAAGCGGACATTG** | **CAGCCATCTCAACGAGTGTGT** | **Vesicle-fusing ATPase** |
| **AK060235** | ***V-ATPase*** | **CTCTGAGGCTCCACTGGGT** | **TCAATCTTCCTCCTCGATGA** | **Vacuolar ATP synthase 91 kDa subunit** |
| **AK099500** | ***ATP-BP*** | **AGACCCAAAGATGCGTCCATC** | **CATCTGCTCGTTGCACTCGCT** | **ATP binding protein** |

[a]Primers are referenced from previous studies[13,14].

**Supplementary Table 8 | Primers for map-based cloning and functional analysis of *OsAAP6*.**

| Name | Type | Forward primer (5'-3') | Reverse primer (5'-3') |
|---|---|---|---|
| RM472 | SSR[a] | CCATGGCCTGAGAGAGAGAG | AGCTAAATGGCCATACGGTG |
| PB1 | InDel[b] | ACTCACTCACTCACCCACACAGC | TTGGAGAGGGAAGAGAAGACACG |
| PB2 | InDel | GTCGAATCGCGATAGTCAGAGC | GGTCAGTTTGGCACTTTGAATGG |
| PB3 | InDel | TGTAGGTAGCTGAACGAGATGAGTGC | CAGGATTGATCCTCATCCCAAGC |
| PB4 | InDel | TCCTGGCTGCTATTGTGGTTGG | AAGTCGTTCGTTGCATGAAGAGG |
| PB5 | InDel | AATCGGCGAGGTTTGCTAATGG | ATACGTGGTACGTGACGCTTTGC |
| PB6 | InDel | AAACTCCTCATTTCGCTCCC | TTGACGAGGCTCTTGTAGGC |
| PB7 | InDel | TCATCGTACAGTGCAGGAG | TCCCTCAGAATCGGCATA |
| PB8 | InDel | CTTCTCACGCCACCTTCC | CTTCCATGCCATTCACCA |
| PB9 | InDel | CCAAGGACCAACTATGACG | GCAGTCTAAGGGAGATAACAAG |
| PB10 | InDel | ACTATCACTGCACTGGGAGC | TGGGGCATGTCATACTGG |
| PB11 | InDel | CGAATCTGGACGGTATTAGGCCGT | GGTTCGAGCTGAATCAGGTAGCAG |
| PB12 | InDel | CATCTAGTAGCTTATTGGTCC | ACCTGCACTTCTCTCTCCCAT |
| PB13 | InDel | ACAGGTCAGGTGCAAAAGCT | AACGCGAACTGGAAACAGAG |
| PB14 | Seq[c] | GCCAAGGCGGAGCTGATCCACCG | CCACGAAGCACACATTGCTACAA |
| PB15 | Seq | GTCGACCAAGGCGTGGCACTT | ACCGATGTTGTACTTCGTTGA |
| RM104 | SSR | GGAAGAGGAGAGAAAGATGTGTGTCG | TCAACAGACACACCGCCACCGC |
| RNAi | | GGCTCACCAAACCTTAAACAA | CTGAGCTACACATGCTCAGGTT |
| A8 | | TCCGCTCAGATAAGAGAGGCCGGAGACTG | CACCAGCAACTAGTACGAGTACAGCAACA |
| Actin1 for qRT-PCR | | TGCTATGTACGTCGCCATCCAG | AATGAGTAACCACGCTCCGTCA |
| GUS | | CCAGGCAGTTTTAACGATCAGTTCGC | GAGTGAAGATCCCTTTCTTGTTACCG |
| Primer for qRT-PCR | | TCTTCCAAACGCAGCTCTGA | CACCAGCAACTAGTACGAGT |
| SCL | | GAATTCTCCGCTCAGATAAGAGAGGCCGGAGACTG | TCTAGAGAGCTGCGTTTGGAAGATGGTGACGTGGC |
| Primer for promoter | | GCCAAGGCGGAGCTGATCCACCG | TGAGAATTACCTGTTCTGACGCG |
| Primer for cDNA | | TCCGCTCAGATAAGAGAGGCCGGAGACTG | CACCAGCAACTAGTACGAGTACAGCAACA |

[a]Simple sequence repeat (SSR) marker.

[b]Insertion/deletion (InDel) marker.

[c]Sequence marker.
